# Women's Menopausal Experiences in the UK: A Systemic Literature Review of Qualitative Studies

**DOI:** 10.1111/hex.70167

**Published:** 2025-01-29

**Authors:** Ailin Anto, Arunima Basu, Rania Selim, Andreas B. Eisingerich

**Affiliations:** ^1^ Faculty of Medicine Imperial College London London UK; ^2^ Imperial College Business School Imperial College London London UK

**Keywords:** menopause, perimenopause, post‐menopausal, UK

## Abstract

**Background:**

Menopause, defined as the cessation of periods for over 12 months, can have a profound impact on women in numerous ways. Understanding women's experiences of menopause can lead to improved care and support during this transformative period.

**Objectives:**

The objective of this systematic review is to identify and summarise published qualitative studies that consider the lived experiences of women with menopause in the UK and to serve as a basis for future research in the field of menopause.

**Search Strategy:**

EMBASE, MEDLINE and PsycINFO databases were searched initially in March, and then updated in April 2024.

**Inclusion Criteria:**

Peer‐reviewed papers with full text available in English, focused on women experiencing menopause in the UK, studies published from January 2000 to April 2024, qualitative or mixed methods study design with qualitative analysis.

**Data Extraction and Synthesis:**

Two authors independently performed title and abstract screening for eligibility. Conflicting opinions were resolved with a third author. Reviewers familiarised themselves with the data and coded the text line by line. Thematic analysis was utilised to place the codes into broader themes. All studies were assessed using an appropriate quality assessment tool.

**Main Results:**

Thirty‐two studies were included in the review with 3462 participants involved. 173 primary codes were extracted and organised into subthemes and 3 overarching themes. These key themes were the biopsychosocial dimensions of menopause, understanding of menopause and strategies to manage menopause.

**Discussion:**

Menopausal experiences documented in the literature are shaped by a range of individual and societal factors. While initiatives to support menopausal women are in place, this review also identifies key knowledge gaps and marginalised groups that would benefit from targeted research and interventions. It emphasises that menopause is not merely a collection of symptoms, but, for many, a pivotal life transition. A deeper understanding of these experiences allows us to more effectively support women through this significant phase of life.

**Conclusions:**

This review concluded that the menopausal experience extends beyond physical symptoms, also affecting mental health, personal and professional life, and self‐identity. Additionally, menopause is shaped by individual life experiences and various personal factors.

**Patient or Public Contributions:**

The studies analysed in this review contain original data from women in the UK undergoing menopause. The qualitative data delves into their experiences with symptoms, accessing various sources of support from NHS and non‐NHS sources as well as alternative therapies.

## 1 Introduction

Within established literature, menopause is typically defined to be the cessation of periods exceeding 12 months [1]. Menopause is often coupled with a spectrum of over 30 recognised symptoms, encompassing hot flushes, mood changes and night sweats [1]. Whilst the average age for women undergoing menopause in the United Kingdom (UK) is 51, the onset can occur typically between the ages of 45 and 55 [2]. Moreover, the duration of the menopausal transition varies considerably among individuals, spanning approximately 7 years on average, though in some cases, it may extend beyond this timeframe [2].

Whilst there has been substantial research into the physical symptoms that arise in women during menopause, over the last few years, there has been a notable increase in our broader understanding of how menopause profoundly influences various aspects of a woman's life. This encompasses recognising the myriads of individual challenges: physical, mental, social and professional, that accompany this stage [3]. The Chartered Institute of Personnel and Development (CIPD) survey reported that over two‐thirds of working women experiencing menopause symptoms have said that it negatively impacts them at work, thus highlighting the economic implications as well [4].

Given that menopause is not merely a biological event but rather a transformative phase that significantly shapes women's day‐to‐day experiences, it is crucial to delve into their lived experiences surrounding it. Experiences are defined by the American Psychological Association to be ‘an event that is actually lived through’ [5]. Experiences are often influenced by the patient's understanding, surrounding environment and beliefs, ethnicity and socioeconomic status which also need to be captured to provide holistic care [6, 7]. Access to participants' lived experiences offers researchers a powerful tool for gaining insight into the meaning of said experiences [8].

Although there is an existing systematic literature review on the experiences of menopausal women worldwide, it dates back several years [9]. Since then, significant shifts have occurred in healthcare practices and perceptions, particularly influenced by the coronavirus disease 2019 (COVID‐19) pandemic. Additionally, given the diverse sociocultural contexts and healthcare systems worldwide, there is a need for a focused systematic literature review specific to the UK. Such a review would offer insights into the unique experiences of menopausal women in the UK.

### 1.1 Objectives

The objective of this systematic review is to identify and summarise published qualitative studies that consider the lived experiences of women with menopause in the UK and to serve as a basis for future research in the field of menopause.

## 2 Methods

### 2.1 Protocol and Registration

The protocol for this review was registered with the International Prospective Register of Systematic Reviews (Prospero; CRD42024536811) [10]. This review was conducted following guidelines from the Preferred Reporting of Items for Systematic Reviews and Meta‐Analyses (PRISMA) statement (Appendix S1) [11].

No patient and public involvement was undertaken. Core outcome set (COS) was not used when planning the systematic literature review.

### 2.2 Design

This is a systematic literature review, which allows for a comprehensive overview of the literature. This enables identification of key findings from other studies, and also highlights any potential gaps within literature.

### 2.3 Search for Key Terms

PICO framework (Population, phenomenon of Interest, and Context) was used to structure the review question.

- Population: Patient/population/problem: UK women experiencing menopause (perimenopausal, menopausal and postmenopausal).
- The phenomenon of Interest: Lived experiences, Experience or Perceptions or Perceive or View or Opinion or Attitude.
- Context/sitting: healthcare and non‐healthcare (home, social networks, occupational).

EMBASE, MEDLINE and PsycINFO databases were searched systematically with help of a health sciences librarian. Table 1 highlights the search terms used, alongside the truncation and Medical Subject Headings (MESH) terms used to capture all relevant studies. The UK filter from Ayiku et al. was utilised to allow for thorough search of published literature in the UK [12]. The initial search was performed in March 2024 and then updated in April 2024.

**Table 1:** A table detailing the search strategy used in this review.

Menopause	Exp menopaus/, ‘menopause and climacterium’/, menopaus*, exp postmenopause/, exp postmenopause/, exp climacterium/, postmenopaus*, Perimenopause*
UK [12]	1. United Kingdom/ 2. (national health service* or nhs*).ti, ab, in, ad. 3. (english not ((published or publication* or translat* or written or language* or speak* or literature or citation*) adj5 english)).ti, ab. 4. (gb or ‘g.b.’ or britain* or (british* not ‘british columbia’) or uk or ‘u.k.’ or united kingdom* or (england* not ‘new england’) or northern ireland* or northern irish* or scotland* or scottish* or ((wales or ‘south wales’) not ‘new south wales’) or welsh*).ti, ab, jx, in, ad. 5. (bath or ‘bath's’ or ((birmingham not alabama*) or (‘birmingham's’ not alabama*) or bradford or ‘bradford's’ or brighton or ‘brighton's’ or bristol or ‘bristol's’ or carlisle* or ‘carlisle's’ or (cambridge not (massachusetts* or boston* or harvard*)) or (‘cambridge's’ not (massachusetts* or boston* or harvard*)) or (canterbury not zealand*) or (‘canterbury's’ not zealand*) or chelmsford or ‘chelmsford's’ or chester or ‘chester's’ or chichester or ‘chichester's’ or coventry or ‘coventry's’ or derby or ‘derby's’ or (durham not (carolina* or nc)) or (‘durham's’ not (carolina* or nc)) or ely or ‘ely's’ or exeter or ‘exeter's’ or gloucester or ‘gloucester's’ or hereford or ‘hereford's’ or hull or ‘hull's’ or lancaster or ‘lancaster's’ or leeds* or leicester or ‘leicester's’ or (lincoln not nebraska*) or (‘lincoln's’ not nebraska*) or (liverpool not (new south wales* or nsw)) or (‘liverpool's’ not (new south wales* or nsw)) or ((london not (ontario* or ont or toronto*)) or (‘london's’ not (ontario* or ont or toronto*)) or manchester or ‘manchester's’ or (newcastle not (new south wales* or nsw)) or (‘newcastle's’ not (new south wales* or nsw)) or norwich or ‘norwich's’ or nottingham or ‘nottingham's’ or oxford or ‘oxford's’ or peterborough or ‘peterborough's’ or plymouth or ‘plymouth's’ or portsmouth or ‘portsmouth's’ or preston or ‘preston's’ or ripon or ‘ripon's’ or salford or ‘salford's’ or salisbury or ‘salisbury's’ or sheffield or ‘sheffield's’ or southampton or ‘southampton's’ or st albans or stoke or ‘stoke's’ or sunderland or ‘sunderland's’ or truro or ‘truro's’ or wakefield or ‘wakefield's’ or wells or westminster or ‘westminster's’ or winchester or ‘winchester's’ or wolverhampton or ‘wolverhampton's’ or (worcester not (massachusetts* or boston* or harvard*)) or (‘worcester's’ not (massachusetts* or boston* or harvard*)) or (york not (‘new york*’ or ny or ontario* or ont or toronto*)) or (‘york's’ not (‘new york*’ or ny or ontario* or ont or toronto*))))). ti, ab, in, ad. 6. (bangor or ‘bangor's’ or cardiff or ‘cardiff's’ or newport or ‘newport's’ or st asaph or ‘st asaph's’ or st davids or swansea or ‘swansea's’). ti, ab, in, ad. 7. (aberdeen or ‘aberdeen's’ or dundee or ‘dundee's’ or edinburgh or ‘edinburgh's’ or glasgow or ‘glasgow's’ or inverness or (perth not australia*) or (‘perth's’ not australia*) or stirling or ‘stirling's’). ti, ab, in, ad. 8. (armagh or ‘armagh's’ or belfast or ‘belfast's’ or lisburn or ‘lisburn's’ or londonderry or ‘londonderry's’ or derry or ‘derry's’ or newry or ‘newry's’). ti, ab, in, ad. 9. or/1‐8. 10. (exp ‘arctic and antarctic’/or exp oceanic regions/or exp western hemisphere/or exp africa/or exp asia/) not (united kingdom/or europe/).
Experience	Experience/, experience*, view*, understand*, attitude, exp attitude to health/, exp attitude/

### 2.4 Inclusion Criteria

The studies included in this review met the following criteria:

1. Studies published from January 2000 to April 2024
2. Peer‐reviewed papers with full text available in English
3. Qualitative or mixed methods study design with qualitative analysis

Only studies focused on the experiences of women and menopause in the UK were included in the systematic literature review. Studies which also explored the experiences of peri‐menopausal women, post‐menopausal women, premature menopausal women and the experiences of women using hormone replacement therapy (HRT) were also included. These terms are defined in Appendix S2 [13, 14, 15, 16].

These terms were also included in this study as often they can formulate a large part of a women's experience of menopause.

### 2.5 Information Sources and Search Methods

#### 2.5.1 Study Selection

Endnote software was used for study selection and removal of duplicates. Two authors independently performed title and abstract screening for eligibility. Conflicting opinions were resolved with a third author. Studies deemed not relevant to the research title, as per the inclusion criteria, were removed at each stage. Post abstract screening, studies that fulfilled the inclusion criteria were read in full and retained if they were deemed to be relevant to the study and still proved to fulfil the inclusion criteria.

#### 2.5.2 Data Extraction

Both authors performed full‐text review and data extraction. Both authors checked the data extraction table to ensure that it was completed in full and completed accurately. Extracted data included (1) publication identification details, (2) study design, (3) population, (4) key findings and (5) limitations. Regarding key findings, the main finding of interest was qualitative data on the lived experiences of women undergoing menopause.

#### 2.5.3 Search Outcomes

**Figure 1:**
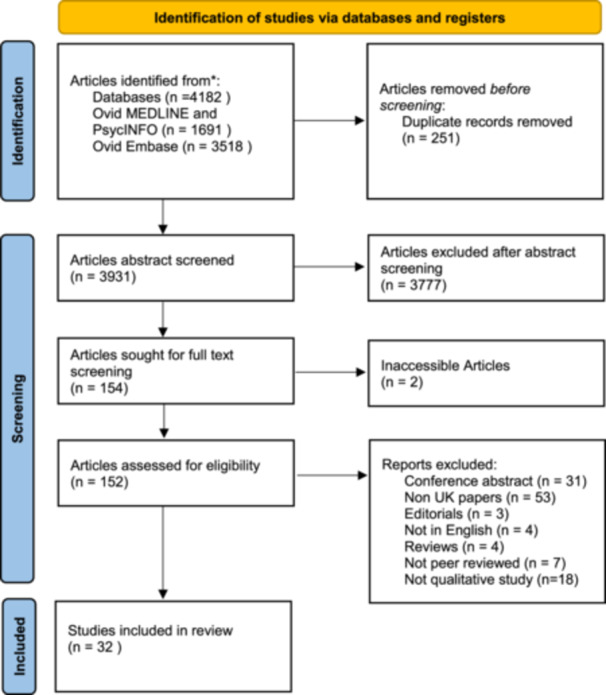
PRISMA Flow diagram to show identification, screening and final included studies [11].

Overall, searching of the databases revealed 3931 results, after 251 removed as duplicates. A total of 32 studies fulfilled the final inclusion criteria and were included in this study, and no additional studies were found through screening reference lists of studies found through our search. Figure 1 (PRISMA Flow Chart) highlights the number of studies obtained at each stage [11].

#### 2.5.4 Quality Assessment of Research Studies

Study quality and applicability were assessed independently by A.B. and R.S. using the Critical Appraisal Skills Programme (CASP) tool [17]. This tool was used as it has a focus on healthcare‐related research and is endorsed by the Cochrane and the World Health Organisation for use in qualitative evidence synthesis [18]. Disagreements were resolved through discussion with A.A. The results of the quality assessment can be found in Appendix S3.

#### 2.5.5 Themes Matrix

A.B. and A.A. familiarised themselves with the data, this involved reading the article carefully and subsequently coded the text line by line. R.S. then reviewed all primary codes, and any discrepancies were resolved through discussion. Thematic analysis was utilised to place the codes into themes by reading through the primary codes and compiling them into relevant groups. The finalised themes were reassessed by going through all the primary codes again to ensure that they were encompassed by the overarching themes adequately.

## 3 Results

### 3.1 Studies Included in the Review

Three databases were searched using the search strategy outlined in Table 1 on 12 April 2024: OVID Medline, EMBASE and PsycINFO. This yielded 3931 results, after 251 were removed as duplicates.

Appendix S4 highlights the characteristics of the studies included and the key conclusions. 32 studies met the inclusion criteria [19, 20, 21, 22, 23, 24, 25, 26, 27, 28, 29, 30, 31, 32, 33, 34, 35, 36, 37, 38, 39, 40, 41, 42, 43, 44, 45, 46, 47, 48, 49, 50], with a total participant count of 3462 women. Of which, 15 studies utilised semi‐structured interviews, 4 used focus groups, 2 used focus groups and interviews and 11 used a mixed methods approach. 19 of the studies focused on the general population, whereas some focused on subgroups such as police workers [33], healthcare workers [19, 22, 24, 28, 41], armed forces workers [48], those with breast cancer [39], intellectual disabilities [26, 43, 49, 50], autism [46] and down syndrome [49, 50].

### 3.2 Key Themes

173 primary codes were extracted from the studies, generating 16 subthemes and 3 overarching themes – the biopsychosocial dimensions of menopause, understanding of menopause and strategies to manage menopause. Each of the three themes is explored below with exemplar quotes. A list of full themes, primary codes, associated studies and illustrative quotes can be found in Appendix S5.

### 3.3 Thematic Analysis

#### 3.3.1 Theme 1: Biopsychosocial Dimensions of Menopause

This overarching theme explores how menopause influences women's physical and mental health, affects their professional and personal relationships, and shapes their social dynamics.

Many menopausal women reported a substantial decline in mental health, along with pronounced shifts in mood and emotions during menopause. Additionally, menopause often intensifies feelings of sadness related to other life events.

I think menopause is just depression really, well it is for me, it just gives you a handful of regrets about everything when you go into the menopause, it's [TOP] something that's lurking in your past that if you're a bit down it comes back at you.

Dykes et al. [44]

Workplace challenges were experienced by most working participants. There was a general lack of support from managers and co‐workers, difficulty in speaking freely about menopause and pressure to adopt a ‘superhero’ mentality in the workplace.

…some of my male colleagues I could just make them shrivel up and die if I started talking about female problems, they would not cope at all well.

MP21, Adelekan‐Kamara et al. [19]

We just plough on … there is that sort of suck it up mentality and just plough on.

MP21, Adelekan‐Kamara et al. [19]

I am not performing as well as I used to. I think that somebody might pick up on it and might criticise me for it. I sort of think to myself ‘I am not getting any younger’. Would they think that maybe they ought to have somebody a bit more on the ball and young? So, it's quite an anxiety.

Griffiths [35]

Some participants experienced a reduction in sex drive, which extended into impacting their relationships. Whereas for others, menopause was associated with a re‐emergence of sexuality.

… My husband didn't have a clue either – bit of an awkward conversation, made me feel washed up and passed it when he's just fine. Feels very unfair. No‐one speaks about the impact on your sex life and these issues have nearly caused me and my husband to separate.

Harper et al. [36]

When I got together with [partner], I just, sort of, to be quite, quite frank, I just said, “The shop's shut, really, I don't think anything works. I am ever so sorry.” He's younger than me, as well, “So I can understand if you want to get your, sort of, exit ticket, I completely understand!” Anyway, bless his heart, he opened the shop, put the awnings out, the whole bloody array of everything on display, and oh my God, I'm having a wonderful time, I've come alive, blimey…. It's bloody good, actually, yeah, I'm hot. Now, that bit doesn't make me feel old!

Salis et al. [42]

Beyond its impact on individuals and their close relationships, menopause significantly affected social dynamics. Many women reported feeling increasingly invisible within society, which negatively influenced their social interactions and often led to increased social withdrawal.

I feel like you become withdrawn from family members, friends, social life, and then there's a worry then, like obviously will people forget about you because you don't want to be this person.

Hobson and Dennis [41]

Women suffer in silence and it is a topic that people don't feel comfortable talking about. It's like you have to hide it as it is so socially unacceptable for women to admit they are getting older!

Harper et al. [36]

#### 3.3.2 Theme 2: Understanding of Menopause

The findings indicated that the participants had specific perspectives regarding menopause, often shaped by the symptoms experienced, their own menstrual experience or mother's experience of menopause.

Because she [her mother] went through it so badly and having expected to be just as volatile, just as moody, just as unpredictable, I've been really happy in the sense of what I go through is not as bad.

Duffy et al. [23]

Many associated menopause with negative connotations, such as a period of loss, commencement of a period of ill health, uncertainty and a devastating event. The concept of stigma associated with menopause was also prominent in many studies. However, some viewed this change as being significantly positive, with ideas of menopause being liberating, indicating a new stage of life, a period of strength and pride and a sense of cleanliness. Whilst others found that some women regarded menopause with relative neutrality, echoing ideas that menopause was a natural stage in life or insignificant to them.

It hasn't been a traumatic change, no, or no big changes … a gentle evolution.

Salis et al. [42]

most horrific time of my life.

Aljumah et al. [32]

It was an embarrassment, maybe it was… there are professional boundaries you just don't want to cross…It is quite intimate isn't it.

MP16, Adelekan‐Kamara et al. [19]

Additionally, their understanding of menopause was shaped by their knowledge. The lack of knowledge regarding menopause was also highlighted through various studies, as well as the broader lack of knowledge around general women's reproductive health.

We aren't informed enough. I'd never even heard the word perimenopause until I spoke to a nurse. I genuinely thought my menopause started when my periods stopped. I've been having symptoms and suffering in silence for 2 years.

Harper et al. [36]

Furthermore, other factors which could impact understanding of menopause were highlighted, with some studies focusing on specific subgroups. One study highlighted improvement of mood secondary to period cessation as a result of menopause in those with learning disabilities. Very few of the qualitative studies identified explored the impact of ethnicity on the perspectives and understanding of menopause in menopausal women.

With autistic girls you need to plan for puberty way before puberty. It's the same with ageing and menopause.

Karavidas and Visser [46]

#### 3.3.3 Theme 3: Strategies to Manage Menopause

The third overarching theme centred around the management of menopause. This revealed complex experiences, highlighting coping mechanisms, treatments and interactions with healthcare professionals (HCPs).

Various coping mechanisms including acceptance and humour were used to manage symptoms. Reframing menopause as an uncontrollable yet normal experience, offered reassurance, especially when comparing their symptoms to others who faced more severe challenges. Peer comparison and shared experiences appeared to reduce stress and enhance resilience.

Those are the women that I go to for support… they just kind of helped me come to terms with that new normal if you like.

(Participant M), Brown et al. [22]

The most reported and important medical treatment was HRT though opinions on its effectiveness were conflicting. Perceptions of HRT were often linked on their knowledge, risk perception and personal health beliefs. Some women found HRT helpful for symptom relief but faced challenges in getting it prescribed.

But she wasn't really willing to start me back on HRT really when the symptoms were so bad …. so eventually, after trying lots of different things, anything prescribable or unprescribable, she had to eventually give me HRT.

Duffy et al. [23]

Others were hesitant to use HRT due to concerns about side effects, cancer risk, difficulty with treatment, and a general lack of information.

… I have no real knowledge about HRT but still feel it's a risky option after the cancer scares of the early 2000s.

Harper et al. [36]

Some studies indicated that women were often informed that HRT was the only available treatment option, leading to a demand for more guidance on alternative therapies. Those who used herbal remedies, vitamins and supplements as well as lifestyle changes found these helped improve quality of life.

So, more time to unwind, more time to go and do nice things‐ that self‐care, like cold water swimming. That's been really helpful, you know. It's‐ it's those things that are going to kind of re‐balance me you know. For me, nutrition and the supplement is really important.

Ray et al. [38]

There were mixed views on the role and effectiveness of HCPs, particularly general practitioners (GPs), in menopause management. While some valued medical support, there was frustration over dismissive attitudes, perceived limitations in expertise, time, and treatment options, driving some women to favour self‐management.

The GP said its natural and I have to put up with it and they can't do anything.

Willis et al. [49]

In terms of priorities obviously it's hard to justify in a stretched primary care service those conditions [menopause] to have the same quality as more serious illness.

Barber et al. [34]

Many preferred consulting older, female GPs, whom they viewed as more empathetic and understanding. Whilst, male GPs were perceived as inclined to dismiss menopausal symptoms. Moreover, there was a strong preference for more detailed information from their GPs, longer consultation times access to female doctors, and better access to specialist care.

Some studies highlighted the variability of diagnosing menopause amongst women. In those with non‐classical symptoms such as mood changes and insomnia, menopause was frequently overlooked, resulting in delayed diagnosis. Studies noted a concerning trend wherein mood‐related symptoms were misdiagnosed as depression and anxiety rather than attributed to menopause. This often led to participants expressing feelings of disappointment with their healthcare provider.

[My GP said] I think you're just depressed because you're not having hot flushes. So, we tried antidepressants. Every time I had my antidepressant review, I brought it up again… I would say, can I just stop you there? I don't think I have an issue [with depression].

Hobson and Dennis [41]

Certain health conditions, including cancer and PCOS, skew the menopausal experience, making symptom differentiation difficult. Many felt that GPs were unable to answer queries about the links between menopause and their comorbidities.

I have PCOS, there is practically no information available about PCOS and the menopause. I have kind of worked out for myself that my more regular periods might be a perimenopause symptom as my excessively high oestrogen levels fall into a more normal range. My GP has absolutely no idea about PCOS and menopause symptoms… I have no idea if there are any extra things I should be trying to manage as the PCOS and menopause interact.

Harper et al. [36]

## 4 Discussion

The collated papers shed light on the diverse menopausal experiences of women in the UK. These experiences are broadly shaped by their menopause, their understanding of menopause and their strategies to menopause management. Although previous reviews have focused on the global population, this review focuses on the UK, a culturally diverse country with residents from a wide range of ethnic backgrounds.

In high‐income countries like the UK, previous studies have found that menopausal experience is shaped by healthcare access, social roles, and family dynamics [9]. The findings of this study corroborate this and found additional factors that affect menopause.

The impact of menopause extends beyond women to their relationships with relationship breakdown being a prevalent issue, attributed to factors such as decreased physical intimacy, lack of supportive partners, and debilitating symptoms [32, 41, 42]. The concept of womanhood and sexuality was also explored. Some saw it as a loss of femininity due to periods stopping [42, 47], while others experienced a resurgence of sexuality and communicated their altered needs to partners [25, 31, 32, 33, 34, 37, 38, 45, 46]. However, challenges like vaginal dryness affected intimacy for some, impacting their sense of femininity and sexuality [29, 32]. Some studies suggest that sexual activity and menopause are separate issues influenced by personality, relationships and health, making it challenging to isolate their relationship [51].

In the UK, approximately 11% of the working population consists of menopausal or post‐menopausal women, with 73% of women aged 40–60 experiencing menopause‐related symptoms during their working lives [52]. As highlighted in this review, these symptoms can be debilitating affecting productivity and attendance [22, 24, 28, 31, 32, 33, 35, 36, 38, 41, 48]. In response to these challenges, the UK government launched the ‘No Time to Step Back: The Government's Menopause Employment Champion’ policy [53], outlining several initiatives aimed at supporting menopausal women in the workplace. Notably, most studies included in this review were published prior to the introduction of this initiative. Future qualitative research should investigate whether these efforts have led to improvements in workplace experiences for menopausal women.

Mental health challenges are prevalent in the UK, with the Office for National Statistics (ONS) [54] reporting that approximately 15% of adults experienced some form of mental illness in 2021. Women, particularly those undergoing menopause, face significant mental health impacts, including heightened anxiety, low mood, and increased stress [22, 23, 25, 26, 27, 31, 32, 36, 38, 41, 42, 43, 44, 46, 47, 49, 50, 54]. This review showed that many menopausal women experience negative body image with a substantial number reporting dissatisfaction with their appearance [24, 26, 32, 33, 37, 45]. This dissatisfaction may be compounded by the cultural emphasis on youth and thinness, which can exacerbate anxiety and depression during menopause [55]. Comparatively, while body image concerns exist globally, the specific pressures in the UK may intensify the emotional challenges faced by menopausal women [56]. In other cultures, perceptions of beauty and body image may be less rigid, potentially offering more diverse standards that can mitigate feelings of inadequacy in Generation Equal [56, 57]. Addressing these body image issues is crucial for supporting the mental health of menopausal women, highlighting the need for a more inclusive representation of women in media and society.

Hoga et al. [9] explore women's global experiences with menopause, identifying consistent themes like feelings of loss, stigma and lack of knowledge, while also noting regional variations. For instance, Hoga et al. found religiosity to be a source of emotional support, a theme less evident in our UK‐focused review, likely due to the country's significant atheist population [58], suggesting that cultural context may shape menopausal experiences.

Our review identifies variation among different subgroups, emphasising how socio‐cultural factors, such as ethnicity, influence menopausal experiences [25]. Notably, ethnic minority women's experiences remain under‐researched in existing literature. This review also underscores the unique challenges for women with medical conditions or intellectual disabilities, who face complexities in diagnosis or misconceptions, respectively, indicating the need for targeted research and support strategies [26, 43, 49, 50]. Although the National Institute for Health and Care Excellence (NICE) guidelines highlight the need for an individualised approach towards the diagnosis, investigation and management of menopause, there is little specific guidance on what this may entail for different subgroups [59].

The literature reveals diverse perspectives on menopause, from neutral to positive or negative, shaped by individual life journeys, as echoed in Hoga et al.'s [9] review [20, 25, 31, 32, 37, 42]. Themes of ageing, loss, and gain are recurrent across studies, reflecting both societal stigma and personal struggles [42, 47]. Feminist scholars argue that longstanding taboos around women's health, including menopause, contribute to feelings of isolation and shame [57, 58]. However, initiatives like Menopause Café UK [60] and peer support can foster openness and provide comfort [19, 22, 23, 25, 26, 28, 32, 37, 38, 41, 46].

A notable finding is the widespread lack of menopause awareness among women, their social circles, and healthcare providers, echoing Zadjali et al.'s findings on osteoporosis in menopausal women [22, 29, 61]. Addressing this knowledge gap could improve health outcomes. Suggestions include mandatory workplace education and incorporating menopause discussions into routine healthcare, such as cervical screenings.

This review also highlights social media's dual role as both a resource and a potential source of misinformation [26, 35, 39]. Unequal access to digital resources further complicates this, with some women, particularly those with intellectual disabilities, preferring non‐digital educational materials [49, 52]. Tailoring resources to diverse needs is essential.

The third overarching theme highlighted the diverse ways in which women navigate menopause, influenced by personal attitudes [19, 22, 23, 25, 26, 28, 32, 37, 38, 41, 46], the availability and perception of treatments [20, 21, 23, 24, 26, 30, 31, 32, 34, 37, 46] and the quality of medical support [22, 23, 27, 32, 34, 36, 41, 46, 48]. Similarly, Hoga et al. found there were varying ways in which women manage their menopause [9]. Symptom management, such as exercise, vitamins and alternative medications, were also deemed as beneficial across studies.

Menopause support in the UK is primarily accessed through the National Health Service (NHS), a system unique to the UK, which shapes distinct experiences of menopause support and care. Our study brings a distinctive focus to the healthcare challenges faced by menopausal women in the UK, highlighting a strong need for structural changes to meet these needs effectively [22, 23, 27, 32, 34, 36, 41, 46, 48]. Our study underscores that some women feel hesitant or lack trust in clinicians' menopause‐related advice, suggesting that alternative avenues for support, outside clinical environments, should be further explored. Additionally, the limited time in GP consultations, coupled with limited access to specialist menopause services, often leaves women without sufficient expertise and guidance [26, 27, 29, 34]. Our findings suggest that enhancing HCP training on menopause, expanding treatment choices, and promoting empathetic, individualised care could substantially improve the menopausal experience for women in the UK.

## 5 Strength and Limitations

To the best of our knowledge, this review is the first to explore the experiences of women undergoing menopause within the UK. This review followed the pre‐published protocol on PROSPERO and adhered to PRISMA guidelines. It benefits from broad search criteria and the use of three databases to increase the chances of finding relevant studies. An appropriate risk of bias tool was used to assess the quality of the included studies.

Due to the explorative nature of menopausal experiences, this systematic literature review only encompassed qualitative studies. While this approach allows for the derivation of ideas and concepts, it does not enable the determination of the strength and frequency of certain experiences. Additionally, only studies in English were included, and it is possible that research undertaken elsewhere may not have been included for this reason. The studies included in this review were often limited by small sample sizes, and some had not yet reached theoretical saturation. Moreover, the studies predominantly involved women who identify as White British, limiting the generalisability of the findings to other ethnic minority groups within the UK population.

## 6 Implications for Clinical Practice

Our review highlights the diagnostic uncertainty faced by those experiencing menopause. A systematic review of guidelines on menopauses [62], found that most guidelines recommend that diagnoses should be symptom‐based, especially in otherwise healthy women aged over 45 years, highlighting the challenges HCPs face in accurately diagnosing the condition. The symptoms of menopause can vary widely, adding to this complexity. This review also points out that women with enhanced knowledge about menopause are better able to self‐advocate. Therefore, increasing awareness and understanding among women, alongside improving symptom recognition among HCPs, can help reduce this uncertainty.

Moreover, the difficulty around understanding and communicating the risks and benefits of HRT in both HCPs and the layman public alike is evident. Guidelines highlight the need to explain the risks and benefits of HRT [59], yet it remains a challenging health topic to counsel on due to the complexity of the information, the negative stigma around HRT on the media and time constraints in the NHS. Furthermore, there is a reluctance among some HCPs to prescribe HRT [63] due to current clinical guidelines focus too heavily on risks and not on the benefits of HRT.

Published literature has found that certain lifestyle changes such as exercise, relaxation, deep breathing [64], incorporation of whole foods, herbal products and soy [65] can improve menopausal symptoms. Alternative therapies have become increasingly popular, yet large gaps exist between patient expectations and HCPs preparedness around alternative therapies. Although the British Menopause Society has highlighted the need to discuss lifestyle and diet modifications in their Top Ten Tips for menopause guidance for practice [66], NICE guidance on menopause only mentions that providing advice that lifestyle changes could help general health and wellbeing [59], rather than making up a formal part of menopause management.

The review points to a lack of qualitative research on the role of ethnicity in menopause experiences within the UK, suggesting this as a valuable area for future study to address current research gaps. This is echoed by the British Menopause Society who published a guide to help clinicians manage women of ethnic minorities undergoing menopause, further stating the menopausal transition in ethnic minority women is poorly understood [67].

## 7 Conclusion

This systematic review offers a comprehensive synthesis of qualitative evidence on the menopausal experiences of women in the UK, emphasising the multifaceted nature of menopause, which spans physical, emotional, and social dimensions. Menopause is not only the end of menstruation; it represents a significant life transition impacting mental health, personal relationships, professional life, and self‐identity. Many women experience declines in mental health, strained professional interactions, and changes in social dynamics, emphasising the pressing need for better support systems.

Our findings highlight that menopause experiences can be greatly improved through enhanced education and healthcare practices. Early education about menopause, across all genders, alongside informed healthcare support, can prepare women for this life phase and address some of the unique challenges they face. By shedding light on these issues and promoting open dialogue, we can foster a society that recognises and supports women through this transformative period, ultimately helping them navigate menopause with greater resilience and well‐being.

## Author Contributions

**Ailin Anto:** conceptualisation, investigation, writing – original draft, methodology, validation, visualisation, writing – review and editing, software, formal analysis, project administration, data curation, resources. **Arunima Basu:** conceptualisation, investigation, writing – original draft, methodology, validation, visualisation, writing – review and editing, software, formal analysis, project administration, data curation, resources. **Rania Selim:** conceptualisation, investigation, writing – original draft, methodology, validation, visualisation, writing – review and editing, software, formal analysis, project administration, data curation, resources. **Andreas B. Eisingerich:** writing – original draft, writing – review and editing, project administration, validation, visualisation, supervision, methodology, investigation, conceptualisation.

## Ethics Statement

The authors have nothing to report.

## Conflicts of Interest

The authors declare no conflicts of interest.

## Supporting information

Supporting information.

## Data Availability

The data that supports the findings of this study are available in the Supporting Information of this article.
