# Supplementary material for: Women's Menopausal Experiences in the UK: A Systemic Literature Review of Qualitative Studies
Source: Health Expect. 2025 Jan 29;28(1):e70167. doi: 10.1111/hex.70167 (PMC11780249; doi:10.1111/hex.70167)
Supplement: Supplementary file 1 — Supporting information. [file HEX-28-e70167-s001.docx]

[S1: PRISMA Flow Chart 1](#_Toc403969987)

[S2: Definitions of Terms 4](#_Toc1183968088)

[S3: Quality Assessment17 5](#_Toc63736714)

[S4: Study Characteristics Table 8](#_Toc1748016337)

[S5: Data Synthesis Table 18](#_Toc1716126584)

# S1: PRISMA Check List

PRISMA Checklist^11^

| **Section and Topic** | **Item #** | **Checklist item** | **Location where item is reported** |
| --- | --- | --- | --- |
| **TITLE** | | |  |
| Title | 1 | Identify the report as a systematic review. | Pg1 |
| **ABSTRACT** | | |  |
| Abstract | 2 | See the PRISMA 2020 for Abstracts checklist. | Pg1 |
| **INTRODUCTION** | | |  |
| Rationale | 3 | Describe the rationale for the review in the context of existing knowledge. | Pg3 |
| Objectives | 4 | Provide an explicit statement of the objective(s) or question(s) the review addresses. | Pg3-4 |
| **METHODS** | | |  |
| Eligibility criteria | 5 | Specify the inclusion and exclusion criteria for the review and how studies were grouped for the syntheses. | Pg4-6 |
| Information sources | 6 | Specify all databases, registers, websites, organisations, reference lists and other sources searched or consulted to identify studies. Specify the date when each source was last searched or consulted. | Pg4 |
| Search strategy | 7 | Present the full search strategies for all databases, registers and websites, including any filters and limits used. | Table 1 |
| Selection process | 8 | Specify the methods used to decide whether a study met the inclusion criteria of the review, including how many reviewers screened each record and each report retrieved, whether they worked independently, and if applicable, details of automation tools used in the process. | Pg5-6 |
| Data collection process | 9 | Specify the methods used to collect data from reports, including how many reviewers collected data from each report, whether they worked independently, any processes for obtaining or confirming data from study investigators, and if applicable, details of automation tools used in the process. | Pg6 |
| Data items | 10a | List and define all outcomes for which data were sought. Specify whether all results that were compatible with each outcome domain in each study were sought (e.g. for all measures, time points, analyses), and if not, the methods used to decide which results to collect. | Pg6 |
|  | 10b | List and define all other variables for which data were sought (e.g. participant and intervention characteristics, funding sources). Describe any assumptions made about any missing or unclear information. | Pg6 |
| Study risk of bias assessment | 11 | Specify the methods used to assess risk of bias in the included studies, including details of the tool(s) used, how many reviewers assessed each study and whether they worked independently, and if applicable, details of automation tools used in the process. | Pg7 |
| Effect measures | 12 | Specify for each outcome the effect measure(s) (e.g. risk ratio, mean difference) used in the synthesis or presentation of results. | N/A |
| Synthesis methods | 13a | Describe the processes used to decide which studies were eligible for each synthesis (e.g. tabulating the study intervention characteristics and comparing against the planned groups for each synthesis (item #5)). | Pg6 |
|  | 13b | Describe any methods required to prepare the data for presentation or synthesis, such as handling of missing summary statistics, or data conversions. | N/A |
|  | 13c | Describe any methods used to tabulate or visually display results of individual studies and syntheses. | Pg6 |
|  | 13d | Describe any methods used to synthesize results and provide a rationale for the choice(s). If meta-analysis was performed, describe the model(s), method(s) to identify the presence and extent of statistical heterogeneity, and software package(s) used. | Pg6 |
|  | 13e | Describe any methods used to explore possible causes of heterogeneity among study results (e.g. subgroup analysis, meta-regression). | N/A |
|  | 13f | Describe any sensitivity analyses conducted to assess robustness of the synthesized results. | N/A |
| Reporting bias assessment | 14 | Describe any methods used to assess risk of bias due to missing results in a synthesis (arising from reporting biases). | N/A |
| Certainty assessment | 15 | Describe any methods used to assess certainty (or confidence) in the body of evidence for an outcome. | N/A |
| **RESULTS** | | |  |
| Study selection | 16a | Describe the results of the search and selection process, from the number of records identified in the search to the number of studies included in the review, ideally using a flow diagram. | Figure 1, Pg7 |
|  | 16b | Cite studies that might appear to meet the inclusion criteria, but which were excluded, and explain why they were excluded. | Figure 1. Pg7 |
| Study characteristics | 17 | Cite each included study and present its characteristics. | Appendix S4 |
| Risk of bias in studies | 18 | Present assessments of risk of bias for each included study. | Appendix S3 |
| Results of individual studies | 19 | For all outcomes, present, for each study: (a) summary statistics for each group (where appropriate) and (b) an effect estimate and its precision (e.g. confidence/credible interval), ideally using structured tables or plots. | Appendix S5 |
| Results of syntheses | 20a | For each synthesis, briefly summarise the characteristics and risk of bias among contributing studies. | Table 1, Appendix S5, Appendix S3 |
|  | 20b | Present results of all statistical syntheses conducted. If meta-analysis was done, present for each the summary estimate and its precision (e.g. confidence/credible interval) and measures of statistical heterogeneity. If comparing groups, describe the direction of the effect. | N/A |
|  | 20c | Present results of all investigations of possible causes of heterogeneity among study results. | N/A |
|  | 20d | Present results of all sensitivity analyses conducted to assess the robustness of the synthesized results. | N/A |
| Reporting biases | 21 | Present assessments of risk of bias due to missing results (arising from reporting biases) for each synthesis assessed. | N/A |
| Certainty of evidence | 22 | Present assessments of certainty (or confidence) in the body of evidence for each outcome assessed. | N/A |
| **DISCUSSION** | | |  |
| Discussion | 23a | Provide a general interpretation of the results in the context of other evidence. | Pg12-15 |
|  | 23b | Discuss any limitations of the evidence included in the review. | Pg15 |
|  | 23c | Discuss any limitations of the review processes used. | Pg15 |
|  | 23d | Discuss implications of the results for practice, policy, and future research. | Pg16 |
| **OTHER INFORMATION** | | |  |
| Registration and protocol | 24a | Provide registration information for the review, including register name and registration number, or state that the review was not registered. | Pg4 |
|  | 24b | Indicate where the review protocol can be accessed, or state that a protocol was not prepared. | Pg4 |
|  | 24c | Describe and explain any amendments to information provided at registration or in the protocol. | N/A |
| Support | 25 | Describe sources of financial or non-financial support for the review, and the role of the funders or sponsors in the review. | Pg2 |
| Competing interests | 26 | Declare any competing interests of review authors. | Pg17 |
| Availability of data, code and other materials | 27 | Report which of the following are publicly available and where they can be found: template data collection forms; data extracted from included studies; data used for all analyses; analytic code; any other materials used in the review. | Appendix |

*Table S1: A table to show where each component of the PRISMA checklist can be found in the manuscript^11^*

# S2: Definitions of Terms

Peri-menopausal: this is often defined as being the stage where the reproductive years of the women is coming to an end. During this transition, the women can experience menstrual irregularities and various other symptoms such as hot flushes^13^.

Menopausal/Post-menopausal: women at the post-menopausal stage have experienced a cessation in their periods for over 12 months^14^. During this phase, the symptoms of menopause can persist, and the hormonal changes experienced during the menopause transition can greatly impact the patient’s health such increasing the risk of cardiovascular disease^14^.

Early menopause: Early menopause is defined to be menopause experienced before the age of 45^15^.

Premature menopause or premature ovarian insufficiency (POI): This is defined to be when menopause occurs before the age of 40^16^.

Hormone replacement therapy (HRT): HRT can come in various forms, but often they all aim to replace the female hormones that decrease during menopause^16^.

# S3: Quality Assessment^17^

| Author | 1 - Was there a clear statement of the aims of the research? | 2 - Is a qualitative methodology appropriate? | 3 - Was the research design appropriate to address the aims of the research? | 4 - Was the recruitment strategy appropriate to the aims of the research? | 5 - Was the data collected in a way that addressed the research issue? | 6 - Has the relationship between researcher and participants been adequately considered? | 7 - Have ethical issues been taken into consideration? | 8 - Was the data analysis sufficiently rigorous? | 9 - Is there a clear statement of findings? | 10 - How valuable is the research? |
| --- | --- | --- | --- | --- | --- | --- | --- | --- | --- | --- |
| Adelekan-Kamara et al ^19^ | Y | Y | Y | Y | Y | N | Y | Y | Y | Valuable |
| Ballard et al ^20^ | Y | Y | Y | Y | Y | N | CT | Y | Y | Valuable |
| Ballard et al ^21^ | Y | Y | Y | Y | Y | N | CT | Y | Y | Valuable |
| Brown et al ^22^ | Y | Y | Y | Y | Y | N | Y | Y | Y | Valuable |
| Duffy et al ^23^ | Y | Y | Y | Y | Y | N | Y | Y | Y | Valuable |
| Green et al ^24^ | Y | Y | Y | Y | Y | N | CT | Y | Y | Valuable |
| Hunter et al ^25^ | Y | Y | Y | Y | Y | N | Y | Y | Y | Valuable |
| Langer-Shapland et al ^26^ | Y | Y | Y | Y | Y | N | Y | Y | Y | Valuable |
| Morris, M. E.  Symonds, A. ^27^ | Y | Y | Y | Y | Y | N | CT | Y | Y | Valuable |
| Prothero et al ^28^ | Y | Y | Y | Y | Y | N | Y | Y | Y | Valuable |
| Utian et al ^29^ | Y | Y | Y | Y | Y | N | CT | Y | Y | Valuable |
| Walter et al ^30^ | Y | Y | Y | Y | Y | N | CT | Y | Y | Valuable |
| Whiley et al ^31^ | Y | Y | Y | Y | Y | N | CT | Y | Y | Valuable |
| Aljumah et al ^32^ | Y | Y | Y | Y | Y | N | Y | Y | Y | Valuable |
| Atkinson et al ^33^ | Y | Y | Y | Y | Y | N | Y | N | Y | Valuable |
| Barber et al ^34^ | Y | Y | Y | Y | Y | N | Y | Y | Y | Valuable |
| Griffiths^35^ | Y | Y | Y | Y | Y | N | CT | CT | Y | Valuable |
| Harper et al ^36^ | Y | Y | Y | Y | Y | N | Y | Y | Y | Valuable |
| Pearce et al ^37^ | Y | Y | Y | Y | Y | Y | Y | Y | Y | Valuable |
| Ray et al ^38^ | Y | Y | Y | Y | Y | N | Y | Y | Y |  |
| Tanna et al ^39^ | Y | Y | Y | Y | Y | N | Y | Y | Y | Valuable |
| Wasley & Gailey ^40^ | Y | Y | Y | Y | Y | N | Y | Y | Y | Valuable |
| Hobson & Dennis ^41^ | Y | Y | Y | Y | Y | N | CT | Y | Y | Valuable |
| Salis et al ^42^ | Y | Y | Y | Y | Y | N | Y | Y | Y | Valuable |
| McCarthy ^43^ | Y | Y | Y | Y | Y | N | Y | Y | Y | Valuable |
| Dykes et al ^44^ | Y | Y | Y | Y | Y | Y | Y | Y | Y | Valuable |
| Rubinstein & Foster ^45^ | Y | Y | Y | Y | Y | N | Y | Y | Y | Valuable |
| Karavidas & Visser ^46^ | Y | Y | Y | Y | Y | Y | Y | Y | Y | Valuable |
| Wellings et al ^47^ | Y | Y | Y | Y | Y | N | Y | Y | Y | Valuable |
| Willmanm & King ^48^ | Y | Y | Y | Y | Y | N | Y | Y | Y | Valuable |
| Willis et al ^49^ | Y | Y | Y | Y | Y | N | Y | Y | Y | Valuable |
| Willis^50^ | Y | Y | Y | Y | Y | N | Y | Y | Y | Valuable |

Table S2: A table to show the results of the risk of bias assessment using the CASP tool^18^.

# S4: Study Characteristics Table

| Title | Author | Year | Study Method | Participant characteristics | Conclusion |
| --- | --- | --- | --- | --- | --- |
| Factors underpinning an improved menopausal experience in the workplace for doctors: a UK-based qualitative study^19^ | Adelekan-Kamara et al | 2023 | Qualitative study: semi structured interviews. Data analysed using thematic analysis. | 41 participants.  Menopausal female doctors (n=21), non-menopausal female doctors (n=6) and non-menopausal male doctors (n=14).  Age range: <30->50 years of age.  Ethnicity: not recorded.  Location: UK. | The study concludes that four key themes could be leveraged to increase menopausal experience in the NHS workplace. These are menopausal knowledge and awareness, openness to discussion, organisational culture and supported personal autonomy. |
| Beyond the mask: women’s experiences of public and private ageing during midlife and their use of age-resisting activities^20^ | Ballard et al | 2005 | Qualitative study: In-depth interviews.  Data analysis used an interpretive approach. | 32 women interviewed from a larger sample of 413 women.  Age range: 51-57 years.  Ethnicity: not recorded.  Location: UK. | This study concluded that menopause was part of 'private ageing' which is less visible to other, however, menopause was viewed as a key indicator to growing older. |
| Understanding risk: women's perceived risk of menopause-related disease and the value they place on preventive hormone replacement therapy^21^ | Ballard et al | 2002 | Qualitative study: audio-taped semi structured interviews.  Data analysis used a phenomenological approach. | 32 women were interviewed from a larger sample of 413 women.  Age range: 51-57 years,  Ethnicity: not recorded.  Location: West Surrey, UK | The study found that women tended to acknowledge menopause with an increased risk of osteoporosis, risk of heart disease. However, influenced by their family history and lifestyle, women do not generally consider themselves to be personally at risk of these diseases. Furthermore, decision to take HRT were primarily determined on their personal judgement of risk. |
| An in-depth qualitative interview study of female ambulance staff experiences of the menopause transition (CESSATION phase 3)^22^ | Brown et al | 2023 | Qualitative study: semi structured interviews.  Data analysed using inductive thematic approach. | 22 female participants  Perimenopause (n = 9); menopause (n = 5); post-menopause (n = 3); and unsure (n = 5). Participants (n=14) had front-line (patient-facing) or emergency operation centre-based roles or were employed in service support roles (n=7).  Age range: 42-62  Ethnicity: not recorded  Location: UK | Ten themes were identified: impact on work role; awareness and preparedness for menopause transition; personal impact of symptoms; desired support; appropriate sickness and menopause policy; managerial development; compassion and dignity; impact of working environment; impact on safety; and lack of choice. |
| The menopause 'It's somewhere between a taboo and a joke'. A focus group study^23^ | Duffy   et al | 2011 | Qualitative study: Four focus groups.  Data analysed using the framework analysis approach. | 14 women participated.  Postmenopausal (n=7), menopausal (n=4), perimenopausal (n=2), unsure (n=1).  Age range: 46-60  Ethnicity: was not recorded.  Location: Grampian region of Scotland. | The key conclusions drawn from this study included some participants feeling less supporting with menopause. They felt that better support networks could help to reduce confusion about the menopausal symptoms and the management options. |
| Narratives of risk: Women at midlife, medical 'experts' and health technologies^24^ | Green et al | 2002 | Interviews | 85 participants.  32 interviews with health professionals and 53 interviews with women were conducted.  Ethnicity: was not recorded.  Location: South Warwickshire and Teesside, UK | The data collected revealed two key themes: social risk and health risk. It concluded that health decisions were influenced by each individuals’ personal situations. |
| Mid-Aged Health in Women from the Indian Subcontinent (MAHWIS): a further quantitative and qualitative investigation of experience of menopause in UK Asian women, compared to UK Caucasian women and women living in Delhi^25^ | Hunter et al | 2009 | Mixed methods study: cross-sectional, quantitative and qualitative interview study.  Qualitative data analysed using framework thematic content analysis. | 153 women  Peri-menopausal and post-menopausal women.  Age range: 45-55  Ethnicity: Three study groups: UK Caucasian, UK Asian and Delhi Asian.  Location: Birmingham, UK and Delhi | The conclusion from the qualitative findings suggested that whilst there were some similarities in key themes between the groups, there were also differences in their experiences of menopause, such as physical symptoms, and the meaning of menopause. |
| It should be more outspoken and not hushed away, not like put in a dark box': An interpretative phenomenological analysis of experiences of menopause voiced by women with learning disabilities ^26^ | Langer-Shapland et al | 2023 | Qualitative study: Semi structured interviews.  Data analysis used interpretive phenomenological approach. | Five women with learning disabilities were interviewed.  Age range: 47-60  Ethnicity: White British (n=4), other unknown (n=1)  Location: South-West England, UK | Five key themes were identified, and these were encompassed under the ideas of information regarding menopause, changes seen, social connection and independent management of menopause. |
| We've been trained to put up with it': Real women and the menopause ^27^ | Morris & Symonds | 2004 | Qualitative study: Focus group initially to inform questions for the semi-structured interviews.  Data analysed using thematic analysis. | 11 women were interviewed. All women worked outside the home.  Age range: not recorded  Ethnicity: White Caucasian  Location: South Wales | The key findings from this paper suggests that women do not have an overall negative view of menopause but are facing challenges with conflicting messages from their own cultural backgrounds, medicalised solutions and pressures of work. |
| Menopause affects us all . . .': menopause transition experiences of female ambulance staff from a UK ambulance service ^28^ | Prothero et al | 2021 | Mixed methods study: utilising a 20-question survey.  Data analysis was carried out using descriptive statistical techniques. Free-text options were thematically analysed using an inductive approach. | 522 participants working in the ambulance service. Participants were either peri menopausal (n=126), menopausal or post-menopausal (n=161), pre-menopausal (n=171) or unsure (n=63)  Age range: not reported  Ethnicity: was not recorded  Location: UK | This study highlights the impact of menopause on the working lives of female ambulance staff in the UK. The free text responses concluded 10 broad themes, the most prominent being lack of menopause awareness and symptom impact. This study brings to light the need for further initiatives to support these women. |
| Attitudes and approaches to vaginal atrophy in postmenopausal women: a focus group qualitative study ^29^ | Utian & Maamari | 2014 | Qualitative study using focus groups.  Data analysed using Censydiam approach. | 70 women participated.  All post-menopausal.  Age range: 40-75  Ethnicity: not recorded  Location: Montreal, Toronto, and Calgary in Canada; Stockholm, Sweden; London, Birmingham, and Manchester in the UK; and New York, Chicago, and San Francisco in the United States. | This study concluded that the attitudes towards vaginal atrophy were driven by personality of the women. |
| Patients' understanding of risk: a qualitative study of decision-making about the menopause and hormone replacement therapy in general practice ^30^ | Walter & Britten | 2002 | Qualitative study using focus groups and semi-structured interviews.  Data was analysed using framework analysis. | 40 women participated  These were current users, ex-users or never users of HRT.  Age range: 50-55  Ethnicity: was not reported.  Location: Cambridge, UK | This study looks at how women understand risk and use that to determine the risk and benefits of using hormone replacement therapy. |
| "A part of being a woman, really": Menopause at work as "dirty" femininity ^31^ | Whiley et al | 2023 | Qualitative study: semi-structured interviews.  Data analysed using interpretative phenomenological analysis. | Six women partook in this study.  All women were mothers.  Age range: not reported  Ethnicity: White-British  Location: UK | This study delves into the understandings of women's lived experience of menopause at work, concluding that some consider menopause at work to be 'dirty'. Two key themes were derived to support the concept of menopause at work as 'dirty' femineity: problematised, pathologised, and “dirty”; and pockets of resistance. |
| An online survey of postmenopausal women to determine their attitudes and knowledge of the menopause ^32^ | Aljumah et al | 2023 | Mixed methods: observational study using an online survey with qualitative component. Qualitative data analysed using thematic analysis. | 594 women answered the qualitative question.  All women were post-menopausal.  Age range 40-56+  Ethnicity: 96.4% participants were Caucasian.  Location: 718 (UK), 111 (other). | Six themes were identified: The need for education, knowledge and understanding of symptoms, why is getting treatment so difficult, feelings and attitudes towards the menopause, the impact of menopause on a woman’s life, the importance of the media –are they getting it right. |
| The menopause taboo at work: Examining women's embodied experiences of menopause in the UK police service ^33^ | Atkinson et al | 2020 | Mixed methods: Quantitative and qualitative data gathered through survey.  Qualitative data analysed using thematic analysis. | 345 women answered the qualitative component. Peri and post-menopausal women recruited from the UK police service.  Age range: 47-59; mean age 50  Ethnicity: 93% of participants were White British.  Location: 3 large police forces, UK | The key codes identified from thematic analysis: experience of symptoms, women’s attitudes and their perceptions of line manager/peer attitudes, and decisions on disclosure. |
| Barriers to Accessing Effective Treatment and Support for Menopausal Symptoms: A Qualitative Study Capturing the Behaviours, Beliefs and Experiences of Key Stakeholders ^34^ | Barber et al | 2023 | Qualitative study: semi-structured interviews. Data analysed using grounded theory approach. | 20 women partook.  All women were post-menopausal.  Age range 47-59.  Ethnicity: 75% of participants were White British ethnicity. 5% of participants were: Indian, Irish, African, white and Black Carribean.  Location: UK | In the cohort of menopausal women barriers to seeking help included: lack of knowledge of menopausal symptoms beyond vasomotor symptoms, misattribution of symptoms to other causes, normalisation of symptoms, stigma and cultural norms regarding menopause in different ethnicities. Barriers to accepting treatment included: Limited information exchange with GPs, perceptions of HRT including risk of cancer, limited information or lack of support regarding deciding about HRT, language used in HRT related consultations. |
| Improving recognition in the UK for menopause-related challenges to women’s working life ^35^ | Griffiths | 2017 | Mixed Methods: qualitative semi-structured interviews. Interviews informed electronic survey. | 60 women.  All women were menopausal  Age range 45-55.  Ethnicity: not reported  Location: UK | Women had mixed experiences of menopause, some finding it more challenging than others. Women found themselves behaving uncharacteristically at work due to menopause and found it difficult to get support from colleagues and managers in the workplace. |
| An online survey of perimenopausal women to determine their attitudes and knowledge of the menopause ^36^ | Harper et al | 2022 | Mixed methods: observational study using an online survey with qualitative component. Data was analysed using thematic analysis. | 545 women answered the qualitative question.  All participants were perimenopausal.  Age range 40-56+.  89.5% of participants were Caucasian.  Other ethnicities: Black/Black British-Caribbean 0.3%, Any other Black/African/Caribbean background 0.3%, Asian/Asian British-Indian 1.4%, Arab 0.1%, Latino 0.3% Mixed ethnic background 1.4%, Any other ethnic group 0.2%  Location: 830 UK, 117 other | Four themes were identified: the overarching knowledge gap, the onset and impact of symptoms, perimenopause: the hidden phenomenon, and managing symptoms: differing schools of thought. |
| Changing Bodies: Experiences of Women Who Have Undergone a Surgically Induced Menopause ^37^ | Pearce et al | 2014 | Qualitative study: text inputted online interviews.  Data was analysed using Interpretive Phenomenological Analysis | 7 women.  All women with surgically induced menopause.  Age range 36-54 and post operation time range 6 months - 19 years.  Ethnicity: White British  Location: UK | Three main themes were identified: the internal body (personal changes the women felt), the meaning of bodily change (hysterectomy symbolised a new stage in life), and the externally judged body (how other people perceived their body). |
| Perimenopausal women's voices: How does their period at the end of reproductive life affect wellbeing? ^38^ | Ray et al | 2023 | Qualitative study: Online focus groups. Data analysed using thematic analysis. | 31 women  All perimenopausal  Age range 40-55.  Ethnicity: 83.8% Caucasian, 12.8% Asian British  Location: UK | This study highlighted that greater education is needed on menstruation involving both genders from a younger age. Women shared key factors for an improved experience includes education of wider population, more support from those around them and the workplace, and better access to information from health care professionals. |
| Exploring the breast cancer patient journey: do breast cancer survivors need menopause management support? ^39^ | Tanna et al | 2011 | Qualitative study: Two in person focus groups.  Data analysed using thematic analysis. | Caucasian women focus group included 13 participants.  7 post-menopausal, 3 perimenopausal, 3 premenopausal.  Age range 49-91.  Asian women focus group included 7 participants. All post-menopausal.  Age range 49-61.  Location: UK | More information that is culturally sensitive on managing menopause symptoms is required. Women require more information on identifying menopausal symptoms compared to side effects of treatment. The study also gives insight into the cultural remedies used for hot flushes, tried by Asian patients |
| Menopause and the role of physical activity – The views and knowledge of women aged 40–65^40^ | Wasley & Gailey | 2024 | Mixed methods. Survey | 162 women and with no serious illnesses. Perimenopausal, menopausal or post-menopausal. Excluded medically induced menopause. Age range 40-65.  Location: UK | Menopause was seen as a taboo subject. Lack of healthcare professional knowledge was a common theme. Most women looked at hormone replacement therapy positively. |
| “I can't be dealing with this brain fog”: A workplace focus group study investigating factors underpinning the menopausal experience for NHS staff ^41^ | Hobson & Dennis | 2023 | Qualitative Study  Structured focus groups  Framework analysis data approach | 14 women  Women working within Welsh NHS services. 7 peri- and 7 menopausal women.  Age Range: 34-59 | Main findings were related to experiences of menopausal symptoms and symptom management, impact of menopause on work and impact of work on menopause. This included disruptive impact of seeking healthcare, negative impact on family and partners, brain fog, sleep disruption and fatigue, anxiety and vasomotor symptoms, support or lack of support from management and peer support. |
| Experiencing menopause in the UK: The interrelated narratives of normality, distress, and transformation ^42^ | Salis et al | 2017 | Qualitative study  Semi structured interviews  Thematic Analysis | 48 women  All British mothers  16 Pre-, 21 Peri- and 11post- menopausal.  Age Range 49-56.  45 British white.  United Kingdom. | Participants described a range of perspectives on menopause, viewing it as a natural transition, a challenging ordeal evoking distress, and a transformative journey. |
| Going through the menopause: perceptions and experiences of women with intellectual disability. ^43^ | McCarthy | 2002 | Qualitative Study  Semi structured interviews  Multi-staged narrative analysis | 15 participants with mild or moderate intellectual disability.  4 pre, 5 peri, 6 post- menopausal.  Age mean: 51 years old (range 43-65).   All white British.  United Kingdom. | Study investigated a wide range of themes including General ageing, Knowledge and expectations: Sources of knowledge, Other knowledge, Physical effects, Women own experiences, Physical changes, Emotional aspects, Support for ID during menopause and Ageing issues in general. |
| Long term follow‐up of emotional experiences after termination of pregnancy: women’s views at menopause ^44^ | Dykes et al | 2011 | Qualitative Study  Semi structured interviews  Template Analysis | 8 women.  Age Mean 47  Age Range 35-63  All were white British.  United Kingdom. | Menopause was considered as a time of reflection over TOP and life. Menopause was viewed as a time of vulnerability to TOP-related negative thoughts, especially where wishes for more children were unfulfilled. However, there were some participants believe menopause had no link to their thoughts on TOP. |
| I don’t know whether it is to do with age or to do with hormones and whether it is do with a stage in your life’: Making sense of menopause and the body ^45^ | Rubinstein & Foster | 2012 | Mixed methods study  Semi Structured Interviews  Thematic Analysis | 12 women.  Peri- and Post menopausal women.  Ethnicity not recorded.  United Kingdom. | Participants reported some negative impacts of menopause involved: Loss of fertility, Loss of attractiveness and becoming “invisible”. The impact of menopause affected participants differently in the level of how they self-objectify themselves. They found a link between negative attitudes towards menopause and heightened body consciousness. |
| “It’s Not Just in My Head, and It’s Not Just Irrelevant”: Autistic Negotiations of Menopausal Transitions ^46^ | Karavidas & Visser | 2022 | Qualitative Study  Individualised interviews  Thematic Analysis | 7 women with autism.  3 Peri- and 4 post-menopausal women.  Age mean 49.4. Age range at 39-63 years.  Ethnicity not recorded.  United Kingdom. | Key themes elicited were the uncertainty about changes, growing self-awareness and self care and navigating support options. Study found there was a limited awareness and understanding of menopause combined with difficulties recognising internal states. Some participants made conscious efforts to resist negative societal constructions of both autism and menopause. Participants when seeing support faced interpersonal and systemic barriers. |
| “We’re Just Tired”: Influences on Sexual Activity Among Male-Partnered Women in Midlife; A Mixed Method Study ^47^ | Welling et al | 2023 | Mixed methods study & qualitative study.  Semi-structured interviews.  Data Analysis using modified Framework Method. | 23 women reporting sexual dissatisfaction in Natsal-3 scale.  Age range: 45-59 | Most salient factor influencing women’s sexual activity in midlife was the complexity of demands on women’s time in this period, and their impact on vitality, rather than physiological decline or hormonal status (I.e. menopause). |
| Serving through the perimenopause: Experiences of women in the UK Armed Forces ^48^ | Willman & King | 2023 | Mixed methods online survey.  Free text data responses.  Iterative thematic analysis. | 465 participants from women working in the armed forces.  Median age was 46 (IQR 42-50).  Ethnicity not recorded.  United Kingdom | Seven themes around menopause were elicited: coping in the workplace, fearing the effect on careers, accessing healthcare, the management of perimenopause by primary care, physical, psychological and cognitive effects, and impact on physical activity. |
| Menopausal Experiences of Women with Intellectual Disabilities ^49^ | Willis et al | 2011 | Qualitative Study.  Semi structured interviews.  Multi-staged narrative analysis. | 45 women with Intellectual Disability, ranging in level from mild to severe.  17 with Downs syndrome and 28 Non downs syndrome.  10 pre-, 15 peri- and 20 post-menopausal women.  Age Range: 35–65 years.  Ethnicity not recorded.  United Kingdom. | Most respondents had a lack of awareness on menopause and menstruation. It was difficult to elicit which behavioural changes (mood swings, low moods) were due to other causes or menopause, there was a need for better health education and more accessible resources. |
| A decade on: what have we learnt about supporting women with intellectual disabilities through menopause ^50^ | Willis | 2008 | Qualitative Study.  Semi-structured interview with pictorial prompts.  Multi-staged narrative analysis. | 15 women with mild to moderate intellectual disability. 4 participants had Downs syndrome.  10 post-menopausal and 5 perimenopausal women.  Ethnicity not recorded.  United Kingdom | Findings revealed little understanding or knowledge about menopause and limited accessible information about the menopause for people with intellectual disabilities. This suggests a need for more accessible information, to increase understanding and awareness of the menopause in these women |

*Table S3: A table detailing the included studies, author name, year of publication, type of study, cohort and key findings.*

# S5: Data Synthesis Table

| Primary Codes (n = 173) | Sub-themes (n= 16) | Overarching Theme | Studies Exploring Themes | Illustrative Quotes |
| --- | --- | --- | --- | --- |
| Participants had symptoms they were not expecting and were shocked | **Symptoms Experienced** | **Theme 1 – Biopsychosocial dimensions of menopause** | Duffy et al^23^, Morris & Symonds^27^, Prothero et al^28^, Aljumah et al^32^, Griffiths^35^, Harper et al^36^, Pearce et al^37^, Ray et al^38^, Tanna et al^39^, Hobson & Dennis^41^, Salis et al^42^ | "‘I sort of doze at my desk in the afternoon … It’s very disconcerting because it’s not like me.’ Griffiths^35^  “Nobody tells you about the joint pain, the muscle ache, the low mood, losing your hair and chronic fatigue, low libido, wanted to throttle everybody in my sight, don't really know what's going on in my head with the brain fog.” - Hobson& Dennis^41^ |
| Negative symptoms impacting daily life; periods becoming unpredictable, feeling physically dirty, insomnia |  |  | Ballard et al 2005^20^, Brown et al^22^ , Duffy et al^23^, Green et al^24^, Hunter et al^25^, Langer-shapland et al^26^, Morris & Symonds^27^, Utian & Maamari^29^, Whiley et al^31^ , Aljumah et al^32^, Atkinson et al^33^, Barber et al^34^, Griffiths^35^, Harper et al^36^,  Ray et al^38^, Tanna et al^39^, Salis et al^42^, Rubinstein & Foster^45^, Wellings et al^47^, Willman et al, Langer-shapland et al^26^, Willis2008^50^, Willis et al 2011^49^, McCarthy^43^, Karavidas & Visser^46^ | “And so that over these…years, I've kind of fluctuated by having the ones that are like a couple of days, to ones that are really heavy, but not like ridiculously heavy, like some women will have to deal with. But it'll just come really, really heavy and then suddenly disappear…or other months have just been really heavy for a long time…and when is this going to end?” [#5] Ray et al^38^     I had awful, like blisters, appearing on my legs. I’ve never had that before, ridiculous. Really, really strange… . I just don’t feel like myself anymore really; - Salis et al^42^ |
| Loss of control of symptoms |  |  | Brown et al^22^ , Green et al^24^, Morris & Symonds^27^, Whiley et al^31^ , Aljumah et al^32^, Atkinson et al^33^, Hobson & Dennis^41^, Salis et al^42^ | “It's so bizarre. I'm laughing because I can be completely fine one minute and then a gibbering wreck the next and it's a bit. It feels to me like it's a guessing game” - Hobson & Dennis^41^ |
| Symptoms resulting in embarrassment or shame |  |  | Brown et al^22^ , Green et al^24^, Langer-shapland et al^26^, Duffy et al^23^; Morris & Symonds^27^, Prothero et al^28^, Whiley et al^31^ , Utian & Maamari^29^, Aljumah et al^32^, Atkinson et al^33^, Barber et al^34^, Hobson & Dennis^41^ | "When I was having heavy vaginal bleeding, I was left by myself. I had a manager just chuck some wipes under the door at me." (Participant F), Brown et al^22^ |
| Symptoms mocked or not taken seriously by others |  |  | Brown et al^22^ , Duffy et al^23^, Prothero et al^28^, Whiley et al^31^ , | "It's a bit of a reoccuring joke that if the older woman complains she's hot she's having a flush" Prothero et al^28^ |
| Sexual symptoms not as commonly mentioned |  |  | Hunter et al^25^, Utian & Maamari^29^ |  |
| Uncertainty on whether symptoms experienced are due to menopause or other causes |  |  | Ballard et al 2005^20^, Brown et al^22^ , Duffy et al^23^, Hunter et al^25^, Langer-shapland et al^26^, Aljumah et al^32^, Barber et al^34^, Harper et al^36^, Tanna et al^39^ | "I didn’t associate them with menopause at the time . . . I thought there was something else wrong with me." (Participant U), Brown et al^22^ |
| Surgical menopause had an instant bodily change |  |  | Aljumah et al^32^, Pearce et al^37^ |  |
| Surgical menopause provided relief for women with pre-existing gynaecological symptoms |  |  | Pearce et al^37^ | “I knew something was wrong” and “I felt unwell. I knew I could not carry on with the way things were.”  “As soon as I had the operation I felt free and could get on with life—back to my sports, playing with the children, not worrying about what I was wearing. And for quite a while I felt amazingly energetic."  Pearce et al^37^ |
| Positive symptoms experienced due to menopause, such as period cessation |  |  | Duffy et al^23^, Hunter et al^25^, Langer-shapland et al^26^, Aljumah et al^32^, Atkinson et al^33^, Barber et al^34^, Harper et al^36^, Pearce et al^37^, Ray et al^38^, Salis et al^42^, , Morris & Symonds^27^, Rubinstein & Foster^45^, Karavidas & Visse, Willis2008^50^, Willis et al 2011^49^ | "Another of the joys of being female but looking forward to not being bothered with periods any more! It’s a beautiful phase of life – like the autumn – a season in life when there is change but moving onto new things! #668" Harper et al^36^    "I’m happier in myself than I’ve ever been in my entire life, which probably sounds odd for you to hear given everything else I’ve been saying… . I’m happy about life and living and happy with the people I work with and happy with the friends I have and, and I think there’s a confidence that comes with age." Salis et al^42^  ‘I am more prepared to speak my mind now’ (6; PeM; LSO) - Rubinstein& Foster^45^  I’m happy about life and living and happy with the people I work with and happy with the friends I have and, and I think there’s a confidence that comes with age. - Salis et al^42^    "no longer having to ‘suffer the inconvenience of periods’ (7; PeM; HSO) - Rubinstein&Foster^45^  “One less thing to worry about” - Karavidas & Visser^46^ |
| Quick diagnosis when symptoms fit classical menopause symptoms | **Variability in Diagnosis** | **Theme 3: Strategies to Manage Menopause** | Barber et al^34^ |  |
| Exploration of other diagnoses before menopause |  |  | Brown et al^22^ , Aljumah et al^32^, Barber et al^34^, Hobson & Dennis^41^ | “Symptoms not recognized by GP, who has prescribed Sertraline for ‘mood symptoms’. I am not taking them, because my low mood reflects hormonal changes, and it is not consistent or like depression. I have requested HRT for current and future health, but the GP has refused it. I have the NICE guidelines to inform GP practice of this peri menopausal gaslighting and mis-prescribing". #163 Harper et al^36^  “[My GP said] I think you're just depressed because you're not having hot flushes. So, we tried antidepressants. Every time I had my antidepressant review, I brought it up again… I would say, can I just stop you there? I don't think I have an issue [with depression]” - Hobson & Dennis^41^ |
| Delay in diagnosis if non classical menopause symptoms |  |  | Barber et al^34^, Harper et al^36^ | “I’ve had so many tests in the last two years for heart palpitations, ectopic beats, etc and nobody suggested this might be caused by the menopause. I’ve suffered from bouts of terrible dizziness. Nobody suggested it might be menopause. My migraines got so much worse I had to see a neurologist, even she didn’t suggest it might be menopause. I had to have a scan because of bloating and abdominal pain, and have terrible adenomyosis, and the radiologist said maybe a coil might help. This led to more discussions around perimenopause incidentally, and now I’m on HRT and finally feel more like me that I have in years. So many doctors and none mentioned menopause once. Yet I was over 45. It’s just wrong”  Harper et al^36^ |
| HCP did not ask about non classical symptoms of menopause | **HCP Practices** | **Theme 3: Strategies to Manage Menopause** | Barber et al^34^ |  |
| GPs advised for HRT as a treatment option |  |  | Aljumah et al^32^, Hobson & Dennis^41^ |  |
| GPs hesitant to prescribe HRT, or doctors advise not to take HRT |  |  | Duffy et al^23^, Aljumah et al^32^, Barber et al^34^, Hobson & Dennis^41^ | "Some women were told by their GP that they were better off without treatment because they would be “introducing a hormone into your body.” Barber et al^34^    “But she wasn't really willing to start me back on HRT really when the symptoms were so bad … .so eventually, after trying lots of different things, anything prescribable or unprescribable, she had to eventually give me HRT." Duffy et al^23^ |
| Incorrect management by health care professionals |  |  | Morris & Symonds^27^, Aljumah et al^32^, Barber et al^34^, Willman&King^48^ , Harper et al^36^, Hobson & Dennis^41^ | !… the wave of multiple symptoms got too much and I went to my gp to ask for HRT. I was shocked at how little she knew, googling things in my appointment and looking up treatments in the BNF. She was very sympathetic however. Unfortunately she prescribed me too high a dose of a combined tablet HRT and within a week I was suffering extreme anxiety and mood changes that forced me to stop. At that point we decided together that my multiple symptoms were too complicated and she referred me to a specialist menopause clinic. When I was seen there the difference in knowledge was staggering and I was so relieved to be given the right HRT. The specialists told me how GP education was organised and I was shocked to hear that menopause is not a compulsory part of their CPD…" Aljumah et al^32^    “[My GP said] I think you're just depressed because you're not having hot flushes. So, we tried antidepressants. Every time I had my antidepressant review, I brought it up again… I would say, can I just stop you there? I don't think I have an issue [with depression]” - Hobson & Dennis^41^    Symptoms not recognized by GP, who has prescribed Sertraline for ‘mood symptoms’. I am not taking them, because my low mood reflects hormonal changes, and it is not consistent or like depression. I have requested HRT for current and future health, but the GP has refused it. I have the NICE guidelines to inform GP practice of this peri menopausal gaslighting and mis-prescribing. #163 Harper et al^36^ |
| Differences in GP based on gender |  |  | Barber et al^34^, Harper et al^36^ | I was treated for anxiety and stress for a long time until I went back to complain about severe night sweats and I was lucky that a youngish woman doctor was on the ball – I had blood tests that confirmed I had hrt patches and coil and within 3 months felt nearly myself again. #566" Harper et al^36^ |
| Dismissive GP attitudes towards Menopause or menopause symptoms |  |  | Duffy et al^23^, Utian & Maamari^29^, Aljumah et al^32^, Barber et al^34^, Willman&King^48^ , Willis et al 2011^49^, Harper et al^36^, Ray et al^38^, Tanna et al^39^, Karavidas & Visser^46^ | ‘The GP said its natural and I have to put up with it and they can’t do anything.’ - Willis et al 2011^49^    "In terms of priorities obviously it’s hard to justify in a stretched primary care service those conditions [menopause] to have the same quality as more serious illness." Barber et al^34^    "I had very little idea of the range of symptoms of the peri-menopause. I knew about hot flushes and I didn’t have those so couldn’t work out what was happening to me. I honestly thought I was going mad. I also really struggled to get any help from my GP – two male GPs dismissed my symptoms and said it’s a phase every woman goes through . . . #" Harper et al^36^ |
| Lack of knowledge of Menopause | **Knowledge** | **Theme 2 – Understanding of menopause** | Adelekan-Kamara et al^19^ , Brown et al^22^ , Duffy et al^23^, Hunter et al^25^, Langer-shapland et al^26^, Prothero et al^28^, Utian & Maamari^29^, Aljumah et al^32^, Barber et al^34^, Harper et al^36^ | "We aren’t informed enough. I’d never even heard the word perimenopause until I spoke to a nurse. I genuinely thought my menopause started when my periods stopped. I’ve been having symptoms and suffering in silence for 2 years.” Harper et al^36^ |
| Lack of preparedness |  |  | Brown et al^22^ , Prothero et al^28^, Aljumah et al^32^, Harper et al^36^ |  |
| Lack of available information and education |  |  | Duffy et al^23^, Aljumah et al^32^, Barber et al^34^, Harper et al^36^, Willis2008^50^  Willis et al 2011^49^ | I consider myself well informed (because in physiology, PhD and work in research) – but I find it hard to know where to look for information. #100 Harper et al^36^ |
| Lack of knowledge on menopause-related disease |  |  | Ballard et al 2002^21^, Morris & Symonds^27^, |  |
| Lack of health care professional knowledge |  |  | Adelekan-Kamara et al^19^ , Utian & Maamari^29^, Aljumah et al^32^, Barber et al^34^, Harper et al^36^, Ray et al^38^, Wasley & Gailey^40^, Willman et al | “And I'd say like 10 years ago, I was obviously having symptoms. And…my GP said, ‘you're too young.’ Right? So, I'm still like, late 30s. I think there's this idea that your symptoms are going to hit you between your mid40s and your mid-50s. And anything outside of that window, anything before mid 40s, it's not perimenopausal, it's something else. And I think that's the education, that knowing you could get symptoms from your mid 30s onwards. And anything that's slightly odd, you know, don't dismiss it as, you know, it could be perimenopause.” [#21] Ray et al^38^ |
| Lack of knowledge amongst general population |  |  | Adelekan-Kamara et al^19^ , Brown et al^22^ , Prothero et al^28^, Aljumah et al^32^, Harper et al^36^, Ray et al^38^, Wasley & Gailey^40^ | "Without a doubt, we need to be teaching children - from teenagers onwards - about the menopause and what it means physically and mentally for those going through it and its various stages. Only by it becoming as normalised as discussing pregnancy will both sexes fully appreciate the impact and effects of it on women (and their partners…)" Aljumah et al^32^    "Men need to be educated too . . . comics need to stop – the suffering is accepted as ‘nagging old moany women’. Pretty sure it would be taken more seriously if men had to go through it too." Harper et al^36^  "It is disappointing the lack of knowledge of many professionals and peers, as such I have had to educate myself. As a headteacher I have created policy and guidelines to make reasonable adjustments for female staff members." Harper et al^36^ |
| Wider lack of knowledge around general women's reproductive health (periods, contraception and menopause) |  |  | Adelekan-Kamara et al^19^ , Ray et al^38^ | "We are so far behind in acknowledging girls have problems." – MP15, Adelekan-Kamara et al^19^ |
| Lack of individual knowledge on HRT |  |  | Langer-shapland et al^26^, Utian & Maamari^29^, Aljumah et al^32^, Barber et al^34^, Harper et al^36^ |  |
| Religion used as information source |  |  | Hunter et al^25^, | "It is written in the Koran that when it starts and when it stops" (DEL21) Hunter et al^25^ |
| Other women, relatives and friends used as information source |  |  | Hunter et al^25^, Aljumah et al^32^, Harper et al^36^ | "I have had more info from colleagues of the same age who have recommended alternatives than anything I have read" Harper et al^36^ |
| Lack of support in the workplace | **Workplace Challenges** | **Theme 1 – Biopyschosocial dimensions of menopause** | Adelekan-Kamara et al^19^ , Brown et al^22^ , Aljumah et al^32^, Atkinson et al^33^, Harper et al^36^, Hobson & Dennis^41^ | "I don’t think there is anything within local trusts to support ladies." – MP1, Adelekan-Kamara et al^19^ |
| Losing job because of menopause |  |  | Brown et al^22^ , Aljumah et al^32^, Willman&King^48^ , | “At the time of my selection [for promotion], I was feeling so low and  suffering from multiple menopause symptoms. I turned down my promotion, was not counselled or checked upon, I felt alone and near to  breakdown. - Willman&King^48^ |
| Concern over symptoms affecting productivity and safety |  |  | Brown et al^22^ , Green et al^24^, Prothero et al^28^, Willman&King^48^ , Hobson & Dennis^41^, Whiley et al^31^, Aljumah et al^32^, Atkinson et al^33^, Griffiths^35^, Harper et al^36^, Ray et al^38^ | “It is just crap. Sometimes seeing the patients … I'm having to  desperately scan through pages and pages of notes to remember who  they are” - Hobson & Dennis^41^  “I need to check I've typed it out correctly so it's just a constant worry and that that adds to my anxiety and my stress” - Hobson & Dennis^41^ |
| Women felt they could not disclose their menopausal status or talk about menopause at work |  |  | Adelekan-Kamara et al^19^ , Brown et al^22^ , Duffy et al^23^, Whiley et al^31^ , Griffiths^35^, Atkinson et al^33^, Willman&King^48^ , Harper et al^36^, Prothero et al^28^, Aljumah et al^32^, | "I wish it wasn’t so taboo and work colleagues/family not roll their eyes when you try and talk about it. #574" Harper et al^36^  "…some of my male colleagues I could just make them shrivel up and die if I started talking about female problems, they would not cope at all well." – MP21, Adelekan-Kamara et al^19^ |
| Pressure of superhero mentality whilst facing menopause challenges |  |  | Adelekan-Kamara et al^19^ , Whiley et al^31^ | "We just plough on … there is that sort of suck it up mentality and just plough on." – MP21, Adelekan-Kamara et al^19^ |
| Feelings of menopause negatively impacting their work and career |  |  | Adelekan-Kamara et al^19^ , Brown et al^22^ , Duffy et al^23^, Green et al^24^, Morris & Symonds^27^, Prothero et al^28^, Whiley et al^31^ , Aljumah et al^32^, Atkinson et al^33^,  Willman&King^48^ ,  Griffiths^35^,  Harper et al^36^,  Hobson & Dennis^41^ | ‘Tiredness, fogginess, concentration issues, irritability and anxiety symptoms all affect my work. I am constantly concerned about what colleagues and bosses must think of me.’ - Willman&King^48^  “How to describe brain fog to your (male) LM [line manager]. Just comes across that you are not working as hard as your colleagues or are not as capable.” - Willman&King^48^  "I am not performing as well as I used to. I think that somebody might pick up on it and might criticise me for it. I sort of think to myself ‘I am not getting any younger’. Would they think that maybe they ought to have somebody a bit more on the ball and young? So, it’s quite an anxiety." Griffiths^35^  "I couldn’t work with the symptoms I was experiencing because I was all over the place, and I had no control on what was going on." (Participant D), Brown et al^22^ |
| Difficulty wearing required work uniforms due to menopausal symptoms |  |  | Brown et al^22^ , Prothero et al^28^, Atkinson et al^33^, Willman&King^48^ , Hobson & Dennis^41^ | “Uncontrollable weight gain adversely affects how an already unflattering uniform looks and consequently how the individual feels. Also having to tuck shirts in/belt combat smocks adds to hot flashes/temperature regulation issues.” - Willman&King^48^   “you take the apron off which has held the sweat in and you literally  look like you've got some sort of like psychedelic pattern on you” - hobson & Dennis^41^  “you can't take it off cause you've got your patient in front of you and I know in the first wave of lockdown we had to wear the full body suit with the visor and everything and I was with this chap once and the perspiration was just like dripping off me. It was in collecting my new gloves which was disgusting” - hobson & Dennis^41^ |
| Women felt more of a need to regulate or keep up an image/appearance in the workplace |  |  | Morris & Symonds^27^, Whiley et al^31^ , Atkinson et al^33^ | "Because you've not got to get old, because you know, you've got to look young" (Diana) Whiley et al^31^ ,    "If you want to be part of a vibrant workforce, you've got to stay young" (Erica) Whiley et al^31^ , |
| Lack of support from other women in the workplace |  |  | Adelekan-Kamara et al^19^ , Atkinson et al^33^, Willman&King^48^ , Harper et al^36^ |  |
| Embarassment to be around male colleagues |  |  | Atkinson et al^33^, Willman&King^48^ , |  |
| Changes in working environment deemed beneficial for managing menopause symptoms |  |  | Brown et al^22^ , Duffy et al^23^, Prothero et al^28^, Griffiths^35^, Ray et al^38^, Hobson & Dennis^41^ | “So, I think from that point of view, supporting you know, like, I work set days in the office because I kind of work with another colleague, and we make sure there's one of us in every day. And sometimes I might have had, you know, might be a heavy day. And it'd be nice not to have to actually go in, be able to have the opportunity to work from home. Because, you know, because of my periods or because of some other perimenopausal symptoms that I'm experiencing.” [#21] Ray et al^38^ |
| Women highlighted the importance of supportive colleagues and managers at work |  |  | Adelekan-Kamara et al^19^ , Brown et al^22^ , Duffy et al^23^, Prothero et al^28^, Ray et al^38^  Hobson & Dennis^41^ | “I'm also extremely lucky to have the line manager and the team that I do…She's in her 30s so she doesn't have the menopause is not on her radar at the moment, but you know in terms of flexibility and if  you're tired start later, finish later. You know, take a break, a longer break if you have to” - Hobson & Dennis^41^ |
| Reducing working hours was deemed beneficial |  |  | Adelekan-Kamara et al^19^ , Prothero et al^28^, Griffiths^35^, Ray et al^38^, Hobson & Dennis^41^ | “and I actually reduced my hours because… I just couldn't cope with life really anymore and I couldn't cope with a full-time job” Hobson & Dennis^41^ |
| Having autonomy over work and flexibility at work was deemed beneficial |  |  | Adelekan-Kamara et al^19^ , Prothero et al^28^, Ray et al^38^, Hobson & Dennis^41^ | “I'm also extremely lucky to have the line manager and the team that I do…She's in her 30s so she doesn't have the menopause is not on her radar at the moment, but you know in terms of flexibility and if you're tired start later, finish later. You know, take a break, a longer break if you have to” - Hobson & Dennis^41^ |
| Feeling supported when working with or being around female dominated workforce |  |  | Atkinson et al^33^ |  |
| Working from home made it easier to deal with unpredictable symptoms |  |  | Brown et al^22^ , Hobson & Dennis^41^ | "Now working from home, it’s much easier to manage. If I’m hot I’ll open a window and not worry about anybody shouting and sort of, not understanding it." (Participant B), Brown et al^22^ |
| Menopause associated with being invisible in society | **Social Dynamics** | **Theme 1 – Biopyschosocial dimensions of menopause** | Utian & Maamari^29^, Harper et al^36^, Rubinstein & Foster^45^, Wellings et al^47^ | "Absolutely need more information and should be talked about more. Women suffer in silence and it is a topic that people don’t feel comfortable talking about. It’s like you have to hide it as it is so socially unacceptable for women to admit they are getting older!" Harper et al^36^ |
| Menopause negatively impacting social engagements |  |  | Green et al^24^, Ray et al^38^, Hobson & Dennis^41^, Wellings et al^47^ | But the problem is I have to sort of earmark my calendar, I can't plan anything. And of course, it's plus or minus two or three days. So basically, it's like a week where I can't…I'm supposed to go on a sailing trip. And it's like, well, I can't be on a sailing trip with my period.” [#^27^] Ray et al^38^  “I feel like you become withdrawn from family members, friends, social life, and then there's a worry then, like obviously will people forget about you because you don't want to be this person” - Hobson & Dennis^41^ |
| Menopause affecting one's ability to fulfil family responsibilities |  |  | Morris & Symonds^27^, Hobson & Dennis^41^  Salis et al^42^, Wellings et al^47^ | “By the time I come home, I'm absolutely shattered. I don't feel as if I got any quality time then for myself or my family because I am basically having a shower, cook some food and then I'm just so shattered” - Hobson & Dennis^41^  What with the kids and all their different issues and our own health issues and trying to keep the house and work. And then obviously parents now as well, they’ve become an issue where they weren’t before, they could help and support, and now we’ve lost one parent that was helpful and supportive and we’ve got others to care for. At this age, you’re in the middle and you’ve got kids at one end and elderly parents that are a bit like kids at the other end. Wellings et al^47^ |
| Patients experienced a massive negative shift in their mental health, mood and emotions during menopause. Menopause exacerbatinng sadness from other midlife events (e.g. children leaving home) | **Mental Health** | **Theme 1 – Biopyschosocial dimensions of menopause** | Brown et al^22^ , Duffy et al^23^, Hunter et al^25^, Langer-shapland et al^26^, Morris & Symonds^27^, Whiley et al^31^ , Aljumah et al^32^, Harper et al^36^, Ray et al^38^  Dykes et al^44^  Salis et al^42^, Hobson & Dennis^41^, Wellings et al^47^, Willman et al, Langer-shapland et al^26^, Willis2008^50^,  Willis et al 2011^49^, McCarthy^43^, Karavidas & Visser^46^ | "My confidence and sense of self worth was on the floor and the tears I have cried, I have lost count of #76" Harper et al^36^    "I think menopause is just depression really, well it is for me, it just gives you a handful of regrets about everything when you go into the menopause, it’s [TOP] something that’s lurking in your past that if you’re a bit down it comes back at you (Ann)" - Dykes et al^44^    "It’s only since the menopause has come on that I actually feel really guilty again, I mean I sometimes sit on my own and I’ve cried because I feel guilty, but I have noticed that has happened more obviously since I’ve gone into the menopause (Elaine)” - Dykes at all |
| Loss of control of temper and having lower patience |  |  | Duffy et al^23^, Hunter et al^25^, Langer-shapland et al^26^, Whiley et al^31^ , Aljumah et al^32^, Harper et al^36^, Ray et al^38^ | “And I had a lot of mood swings. I became so anxious at any slight provocation. I was very uncomfortable.”  Ray et al^38^  "…I was ridiculously short tempered and sometimes violent - I slapped my children…" #880 Aljumah et al^32^ |
| Sadness as losing ability to have children |  |  | Harper et al^36^, Pearce et al^37^, Salis et al^42^, Dykes et al^44^, Rubinstein & Foster^45^ | f I had been more aware of what the symptoms were, and had had more of an understanding from my GP when I went with increasingly heavy/clotting periods at 40/41, then I feel I would have realised what was happening and looked to treat those symptoms much earlier. I have found it particularly difficult, not having children, as you realise that the whole reason for women having the presence of a menstrual cycle is to be fertile and produce children. So knowing that it is all going to end without feeling it was ‘useful’ in any way is hitting quite hard. #759 Harper et al^36^   "It’s like part of you has gone, when you go through the menopause, part of you being a woman what’s gone, reproducing, I think it’s quite sad that I can’t have any more children" - Dykes et al^44^    “sad feeling with the finality of not being able to have another child.” Pearce et al^37^ |
| Being treated with dignity positively impacted wellbeing |  |  | Brown et al^22^ , Prothero et al^28^, |  |
| Negative perception of body image |  |  | Green et al^24^, Langer-shapland et al^26^, Aljumah et al^32^, Atkinson et al^33^, Pearce et al^37^, Rubinstein & Foster^45^ | "If I feel fat I feel rubbish [bad]. If I feel my body image is good then I feel good.” Pearce et al^37^ |
| Importance of emotional support |  |  | Ray et al^38^, Wellings et al^47^, Morris & Symonds^27^, | “The most support I need…is the emotional support…I'm quite emotional. So…during my period, I enjoy emotional support. Ray et al^38^ |
| Feeling of loss of youth causing anxiety |  |  | Whiley et al^31^ , Ray et al^38^, Rubinstein & Foster^45^ | “The way it has affected me is that my social skills have like really gone down because…I'm seeing young girls walk around… it just dawns on me, “Okay, I've passed through this phase like…I no longer get to ovulate any longer, get to see the whole normal period thing,” and somehow it has really affected the way I-I socialise with people because…I don't know, to some extent I just tend to look down on myself. I know it's a normal adult like…adult women thing. But then it…just really doesn't give me the space to like express myself…” [#8] Ray et al^38^ |
| Menopause caused relationship breakdown | **Impact on sex and relationships** | **Theme 1 – Biopyschosocial dimensions of menopause** | Aljumah et al^32^, Harper et al^36^, Wellings et al^47^ | It’s impacted my relationship with my husband and I’m now battling to save my marriage all because of perimenopause!! #7^34^ Harper et al^36^  ". . . My husband didn’t have a clue either – bit of an awkward conversation, made me feel washed up and passed it when he’s just fine. Feels very unfair. No-one speaks about the impact on your sex life and these issues have nearly caused me and my husband to separate." Harper et al^36^ |
| Menopause symptoms affecting partner |  |  | Aljumah et al^32^, Hobson & Dennis^41^  Salis et al^42^ | “…I said, ‘hopefully now I can start to get my mojo back’ and he was just like ‘I would love that’. And I thought, wow, that's when I realised how much it affected him” - Hobson & Dennis^41^ |
| Vaginal dryness making intercourse difficult |  |  | Utian & Maamari^29^, Aljumah et al^32^ |  |
| Reduced sex drive |  |  | Utian & Maamari^29^, Aljumah et al^32^, Pearce et al^37^, Wellings et al^47^ | "“All I had inside me was a black hole that made me feel strange,” and she believed this stopped her from wanting to “make love." Pearce et al^37^ |
| Vaginal atrophy associated with a reduction in the feeling of feminity |  |  | Utian & Maamari^29^, |  |
| Partner lack of interest |  |  | Wellings et al^47^ | I think his fear of failure, financially, you know, we are as I said less than secure despite this beautiful house, huge mortgage, never going to be repaid, no pension. That worries him, he can’t cope. It’s classic really … if he hits any sort of professional hiccup or he feels challenged, it’s not that he doesn’t enjoy sex but that he hides, he literally crawls under the duvet, he can sleep all weekend, he cannot get out of bed. [8] Wellings et al^47^ |
| Reemergence of sexuality and transformation of relationship |  |  | Salis et al^42^, Wellings et al^47^ | When I got together with [partner], I just, sort of, to be quite, quite frank, I just  said, “The shop’s shut, really, I don’t think anything works. I am ever so sorry.”  He’s younger than me, as well, “So I can understand if you want to get your, sort  of, exit ticket, I completely understand!” Anyway, bless his heart, he opened the  shop, put the awnings out, the whole bloody array of everything on display, and oh  my God, I’m having a wonderful time, I’ve come alive, blimey… . It’s bloody good,  actually, yeah, I’m hot. Now, that bit doesn’t make me feel old! - Salis et al^42^  “I know there’s always an  outside chance that you could fall pregnant but, the likelihood is I won’t, you  know, so I’m looking to, or we are looking to you know, grow into each other  again. -” Salis et al^42^  “. . . the  chances of getting pregnant are virtually nil aren’t they? So from  that point of view I look at it in a positive light.” - Wellings et al^47^ |
| Menopause not linked to sexual activity, frequency or quality |  |  | Wellings et al^47^ |  |
| Personality types were associated with different ways of managing vaginal atrophy |  |  | Utian & Maamari^29^ |  |
| Sharing experiences with others was deemed beneficial, particularly with close family members, at the work place, with some health staff and those who has themselves experienced menopause | **Coping Mechanisms** | **Theme 3: Strategies to Manage Menopause** | Adelekan-Kamara et al^19^ , Brown et al^22^ , Duffy et al^23^, Hunter et al^25^, Langer-shapland et al^26^, Prothero et al^28^,Aljumah et al^32^  Pearce et al^37,^ Ray et al^38^,  Karavidas & Visser^46^, Hobson & Dennis^41^ | "Women find it quite positive to have the fact that it’s acknowledged and talked about." – MP17, Adelekan-Kamara et al^19^ . "Those are the women that I go to for support... they just kind of helped me come to terms with that new normal if you like." (Participant M), Brown et al^22^  "She found it useful to “turn to others for support from partners, friends, and other women with similar experiences.” She expressed the importance of “knowing you are not the only one.” Pearce et al^37^  It’s not just in my head and it’s not just irrelevant. Sometimes I think I’m just making a fuss, but then when I to talk to someone else they are like ‘I have the same thing’. - Karavidas & Visser^46^ |
| Women compared themselves to other women who were worse off as a coping mechanism |  |  | Pearce et al^37^, Salis et al^42^ | All women comparing to women who were “worse off” Pearce et al^37^ |
| Mental acceptance of menopause and it's symptoms was used as a coping mechanism |  |  | Pearce et al^37^, Salis et al^42^ |  |
| Using humour to deflect |  |  | Brown et al^22^ , Pearce et al^37^ | “laughing it off” and “not dwelling on it.” Pearce et al^37^ |
| Using concealment |  |  | Pearce et al^37^, Salis et al^42^, Morris & Symonds^27^, Whiley et al^31^ , Harper et al^36^ | “dressing down to hide in the crowd,” Pearce et al^37^ |
| Pushing through the discomfort mentality/Tolerate the changes |  |  | Karavidas & Visser^46^, Salis et al^42^ | “It’s been a bit of a theme to my life that I tend to push on through”. - Karavidas & Visser^46^ |
| Supportive partner and family |  |  | Wellings et al^47^, Duffy et al^23^, Langer-shapland et al^26^, Tanna et al^39^ |  |
| Menopause was seen as a devastating event in life | **Perspective on Menopause** | **Theme 2 – Understanding of menopause** | Aljumah et al^32^ | "most horrific time of my life" Aljumah et al^32^ |
| Menopause was seen as insignificant or not a dramatic change |  |  | Hunter et al^25^, Whiley et al^31^ , Salis et al^42^ | I don’t think those physical aspects of your life define anything in life, actually… .It’s the other things, it’s your, you know, intellectual, personal and emotional development that defines who you are, not specific biological things. - Salis et al^42^  “It hasn’t been a traumatic change, no, or no big changes … a gentle evolution.” - Salis et al^42^ |
| Menopause primarily defined by the symptoms experienced |  |  | Duffy et al^23^, Langer-shapland et al^26^, |  |
| Commencement of future ill health |  |  | Hunter et al^25^, Morris & Symonds^27^, Whiley et al^31^ , Salis et al^42^ | "God knows when periods stop it is difficult for women, you get hundred of illnesses" (UKA51) Hunter et al^25^, |
| Menopause as a mid-life marker and a new phase of life/transformation |  |  | Ballard et al 2005, Hunter et al^25^, Pearce et al^37^, Salis et al^42^ | "It just means you’re going from one phase in your life to another.” Pearce et al^37^ |
| Menopause seen as a period of strength and pride |  |  | Utian & Maamari^29^ |  |
| Sense of disconnect between their mental and physical health status |  |  | Utian & Maamari^29^, Aljumah et al^32^ | "…My mind and body feel like strangers. It has been awful." Aljumah et al^32^ |
| Menopause seen as an excuse |  |  | Duffy et al^23^, Morris & Symonds^27^, | "It's kind of like, it's somewhere between a taboo and a joke and you kind of can't have a proper conversation about it … But it's almost like there's something about not wanting to say you're menopausal because it's kind of viewed as an excuse for something … yea it's maybe not taken seriously, there's a whole lot of funny things wrapped up in it." Duffy et al^23^ |
| Menopause was associated with ageing and being old |  |  | Ballard et al 2005^20^, Hunter et al^25^, Morris & Symonds^27^, Whiley et al^31^ , Aljumah et al^32^, Atkinson et al^33^,  Barber et al^34^, Harper et al^36^, Salis et al^42^ Ray et al^38^,  Rubinstein&Foster^45^ 2012, Wellings et al^47^, Karavidas & Visser^46^, McCarthy^43^, Willis et al 2011^49^, Willis2008^50^ | it’s the beginning of dying, and I kind of think it is really because I am quite sort of family centric…. It’s an opportunity to go out and have more coffees with friends and things, but actually I’m quite sad about it as well and quite worried about this next stage of my life. - Salis et al^42^   I feel like my life’s over, and it’s too late to start a new career, not that I want a new career, but it’s too late to attract men… . Beginning to get wrinkly and not being fertile anymore and not, not being needed as a mum, you know, the adoration. It marks the end of being young and attractive and fertile. And it’s a mark of the beginning of old age. I just feel like an old hag. That’s how I feel inside, so I just feel a has-been. - Salis et al^42^ |
| Menopause seen as a time of liberation |  |  | Whiley et al^31^ , Aljumah et al^32^, Atkinson et al^33^, Barber et al^34^, Harper et al^36^, Pearce et al^37^, Ray et al^38^, Rubinstein&Foster^45^, Salis et al^42^ | Some women’s sense of selfhood depends on this aspect, but not for me. I can be myself again because I am freer, and that in turn is liberating. I do not feel the same as I did before: I feel better. Pearce et al^37^    new stage of creativity where I am moving to a more mature stage of life - Rubinstein & Foster^45^ |
| Menopause seen as a natural period in a woman's life |  |  | Adelekan-Kamara et al^19^ , Brown et al^22^ , Hunter et al^25^, Morris & Symonds^27^, Whiley et al^31^ , Aljumah et al^32^, Atkinson et al^33^, Barber et al^34^, Rubinstein&Foster^45^  Salis et al^42^ | it was an ‘inevitable step’ (1; PeM; HSO) and ‘an important and natural milestone’ (3; PeM; HSO) - Rubinstein & Foster^45^ 2012  "It’s like childbirth, you know. Well it’s natural, it’s not like a terrible thing that strikes you, you know, it’s not an illness or, and you’ve got to just go through it in a way… . I’ve been very lucky as well, not been affected hardly at all."- Salis et al^42^ |
| Menopause has been over medicalised |  |  | Morris & Symonds^27^, Aljumah et al^32^, Harper et al^36^, Karavidas & Visser^46^ | "I feel offended by the implication that menopause is a hormone deficiency syndrome. I think this is a misogynistic, patriarchal view even though it is often perpetuated by women. It is a difficult life stage for many people, but a natural process and women should not be regarded as deficient or broken in some way. #50" Harper et al^36^ |
| Some women had the mentality that they should just deal with it and get on with it |  |  | Brown et al^22^ , Hunter et al^25^, Langer-shapland et al^26^, Morris & Symonds^27^, Barber et al^34^, Salis et al^42^ | "I just plodded on, kept my mouth shut and just got on with it." (Participant I) Brown et al^22^ |
| Menopause seen as a loss and ending: loss of feminity including loss of fertility, loss of being a woman, loss of attractiveness, and loss of sexual availability |  |  | Hunter et al^25^, Whiley et al^31^ , Aljumah et al^32^, Atkinson et al^33^, Barber et al^34^,  Pearce et al^37^,  Ray et al^38^,  Rubinstein&Foster^45^  Salis et al^42^ , Karavidas & Visser^46^ | "“All of your other bodily changes signify expectancy (puberty, pregnancy), whereas menopause signals a closing down of purpose.” Pearce et al^37^    I could usually attract people and whatever and then suddenly you virtually wake up and you are old and ugly … - Rubinstein&Foster^45^   Yes, I’m sad in the sense to acknowledge the fact that you are now of an age where having more children – that it’s not your turn any more – you know the younger generation’s turn.(1; PeM; HSO) - Rubinstein&Foster^45^ 2012    it is incompatible to express a sexual kind of wolf whistle public behaviour or even think those thoughts with someone who is menopausal or postmenopausal. Because it is like saying that you want to do it with your grandmother or mother. It is not natural and it is not proper. So I think there are certain imperatives about nature and the way nature works around fertility and reproduction that dictate human fundamental behaviour. (3; PoM; HSO) - Rubinstein&Foster^45^    I felt like I gave out this vibe of not being sexy anymore, and I really do think there  is a difference… . I think there must be something about women who are ovulating  that’s, you know, subliminally attractive or attracts men. - Salis et al^42^ |
| Sense of uncertainty |  |  | Hunter et al^25^, Whiley et al^31^ |  |
| Increased sense of cleanliness |  |  | Hunter et al^25^, Langer-shapland et al^26^, | "Good dirty business is over" (UKA15), "feel clean now no mess" (UKA63) Hunter et al^25^, |
| Women did not think about menopause until it happened to them |  |  | Griffiths^35^ |  |
| Perspectives/experience of menopause influenced by own menstrual experience or mothers experience or other women in their lives' experience of menopause |  |  | Duffy et al^23^, Morris & Symonds^27^, Salis et al^42^ | "Because she [her mother] went through it so badly and having expected to be just as volatile, just as moody, just as unpredictable, I've been really happy in the sense of what I go through is not as bad." Duffy et al^23^ |
| Menopause affected one's identity and selfhood |  |  | Duffy et al^23^, Aljumah et al^32^, Barber et al^34^, Salis et al^42^ |  |
| There is a link between high self-objectification and negative feelings towards menopause |  |  | Rubinstein&Foster^45^ 2012 |  |
| Menopause seen as a taboo/stigma/embarassing subject |  |  | Adelekan-Kamara et al^19^ , Brown et al^22^ , Duffy et al^23^, Hunter et al^25^, Langer-shapland et al^26^, Morris & Symonds^27^, Whiley et al^31^ , Aljumah et al^32^,  Atkinson et al^33^, Barber et al^34^, Harper et al^36^, Karavidas & Visser^46^, Salis et al^42^ | It was an embarrassment, maybe it was… there are professional boundaries you just don’t want to cross…It is quite intimate isn’t it. – MP16, Adelekan-Kamara et al^19^ |
| Better access to specialist clinics or GPs specialising in menopause | **Suggestions for Improved Experience** | **Theme 3: Strategies to Manage Menopause** | Aljumah et al^32^, Willman&King^48^ , Tanna et al^39^ | “It is difficult to communicate and understand your symptoms when so  little is known about the menopause [by HCPs], especially when medical  professionals have to look up details as they don’t routinely deal with the  older generation. It is almost like a hidden disability. More training or the  ability to access the specialist.” - Willman&King^48^ |
| Better access to reliable information on menopause and its management |  |  | Duffy et al^23^, Langer-shapland et al^26^, Prothero et al^28^, Aljumah et al^32^, Griffiths^35^, Harper et al^36^, Ray et al^38^, Tanna et al^39^, Barber et al^34^ | "If we were provided with advice about how to reduce symptoms such as exercise or walking, I would have had more motivation to do these things." Harper et al^36^ |
| Desire for more awareness and openness for discussion on menopause in the workplace |  |  | Adelekan-Kamara et al^19^ , Brown et al^22^ , Morris & Symonds^27^, Aljumah et al^32^, Griffiths^35^, Harper et al^36^, Ray et al^38^, Hobson & Dennis^41^ | “It's a very understanding team and there's a lot going through the  same. So we're all yeah sympathising with each other” - Hobson & Dennis^41^. |
| Desire for better support in the workplace such as from managers and colleagues, mandatory training and menopause policies |  |  | Adelekan-Kamara et al^19^ , Brown et al^22^ , Morris & Symonds^27^, Prothero et al^28^, Aljumah et al^32^, Ray et al^38^ | "I think workplace policies would help so you don’t feel guilty of taking time off if you had to." – MP10, Adelekan-Kamara et al^19^ |
| Desire for flexible working hours |  |  | Griffiths^35^, Ray et al^38^, Hobson & Dennis^41^ |  |
| Adjustments to the working environment, Better temperature control in the workplace |  |  | Adelekan-Kamara et al^19^ , Brown et al^22^ , Prothero et al^28^, Griffiths^35^ |  |
| Desire for informal support, such as support groups and telephone advice line |  |  | Morris & Symonds^27^, Prothero et al^28^, Aljumah et al^32^, Griffiths^35^, Ray et al^38^ | "I wish there was a chance to have menopause and peri menopause discussed in women’s groups before it happens and as it happens" Aljumah et al^32^, |
| Educating males, including children and partners |  |  | Brown et al^22^ , Aljumah et al^32^, Harper et al^36^, Ray et al^38^ | “I think I think the education about perimenopause and the menopause needs to be started, you know, with school age children. So they understand what their par- what their mums are going through…but to do it in such a way that doesn't frighten them as well and…just thinking about ways in which it can be done in a positive way to empower women, and the men that are around them, to feel like they know what's happening and when to you know, seek, seek support, I think would be would be really, really helpful.” [#5] Ray et al^38^ |
| Integrating menopause into routine health check ups |  |  | Harper et al^36^ |  |
| Better training for healthcare professionals on menopause, including during medical school |  |  | Adelekan-Kamara et al^19^ , Willman&King^48^ , Ray et al^38^, | “It is difficult to communicate and understand your symptoms when so  little is known about the menopause [by HCPs], especially when medical  professionals have to look up details as they don’t routinely deal with the  older generation. It is almost like a hidden disability. More training or the  ability to access the specialist.” - Willman&King^48^    “And I also think training healthcare providers, enhancing medical training programs to include comprehensive education on menopause and perimenopause could also go a long way to help, this will enable healthcare providers to better understand the symptoms and the treatment options, and then appropriate management strategy.” [#15] Ray et al^38^ |
| No change at work needed |  |  | Adelekan-Kamara | "However, this belief was not echoed by all NMP’s, as one non-MP believed not much support was required" (NMP8) Adelekan-Kamara, |
| Better education in schools |  |  | Aljumah et al^32^, Harper et al^36^, Ray et al^38^ | “This should be taught at school along with menstrual cycle in full detail.” Aljumah et al^32^ |
| Increased access to period products in schools to normalise periods |  |  | Ray et al^38^ | “I'm also thinking… if there's proper access to menstrual products, you know, given in schools…it's to make the teenage girls know that “Okay, these things are things that we need. These things are not…weird. They are things that are actually important for my hygiene. So, I should not be ashamed of going to the grocery shop to get these things, to the supermarkets to get the pads, the tampon, you know.” Ray et al^38^ |
| Conflicted opinions on the effectiveness of HRT | **Management** | **Theme 3: Strategies to Manage Menopause** | Duffy et al^23^, Aljumah et al^32^, Barber et al^34^ |  |
| Judging risk of HRT depended on multiple personal factors and core beliefs |  |  | Ballard et al 2002^21^, Ballard et al 2005^20^, Green et al^24^, Walter & Britten^30^, |  |
| HRT used for age resistance |  |  | Ballard et al 2005^20^ | I think that by maintaining a fairly healthy lifestyle you can aim to offset theravages of old age as much as possible. And I think that nowadays, there’s somuch on the market to help you – exercises and gadgets, and home gymnasiums.I think that HRT does help as well. Well, it certainly helped me to come to termswith getting old. It [HRT] continues to make you feel . . . your skin feels betterand you have a bit more energy and feel more alive, and that helps you retaina bit of a youthful appearance.”  Ballard et al, 2005 |
| HRT used for symptom relief, with increased symptom severity associated with increased likelihood of uptake |  |  | Duffy et al^23^, Green et al^24^, Walter & Britten^30^, Whiley et al^31^ , Aljumah et al^32^, Willman&King^48^ , Karavidas & Visser^46^ | I was always one of those people who was like ‘No, I’m not going to take hormones, it’s all a natural process’…and now I’m sitting here and going ... I haven’t slept through the night in months” (Olivia). - Karavidas & Visser^46^ |
| HRT used to prevent menopause-related conditions |  |  | Ballard et al 2002^21^, Duffy et al^23^, Green et al^24^, Langer-shapland et al^26^, Aljumah et al^32^ | "The blood tests showed that some months I was definately menopausal and other months I showed no signs. But they [doctors] weighed it up and said, well looking at this [hormone levels] and looking at your build (becuase I was very thin)...they said that I would be an ideal wheelchair candidate if I didn't go on HRT" (R13), Ballard et al 2002^21^ |
| Those medically 'diagnosed' with menopause-related conditions likley to stay on HRT, but weighing up the advantages and disadvantages |  |  | Ballard et al 2002^21^, Green et al^24^, | "No, no I don't see that I will stop it [HRT]. I mean what you read sometimes makes you feel, oh dear this is a worry to be on it. but the people that I have spoken to...consultants etc. who have spoken to me about it, I feel have got to know and I would take their advice...Regardless to what's been read"  Ballard et al 2002^21^ |
| Desire for individualised/holistic menopause management |  |  | Duffy et al^23^, Langer-shapland et al^26^, Harper et al^36^, Karavidas & Visser^46^ |  |
| Not at personal risk of menopause related conditions, thus not likely to take HRT as prevention |  |  | Ballard et al 2002^21^ | "I drink 4 pints of milk a day and have done since I was a kiddy...Well I'm not going to get osteoporosis. I know that." (R20), Ballard et al, 2002 |
| Negative experience with HRT |  |  | Ballard et al 2002^21^, Duffy et al^23^, Green et al^24^, Langer-shapland et al^26^, Walter & Britten^30^, Pearce et al^37^, Salis et al^42^, Karavidas & Visser^46^ | "“It makes me feel awful, depressed, fed up, frumpy. Before, I was really petite, skinny, and energetic.” Pearce et al^37^  I woke up one day and said, ‘I can't live like this! it's making me brain dead!’” (Dora). Karavidas |
| Hesitancy to commence HRT due to risk profile and lack of information |  |  | Ballard et al 2002^21^, Ballard et al 2005^20^, Duffy et al^23^, Green et al^24^, Langer-shapland et al^26^, Aljumah et al^32^  Barber et al^34^,  Harper et al^36^  Hobson & Dennis^41^ | ". . . I have no real knowledge about HRT but still feel it’s a risky option after the cancer scares of the early 2000’s. #^46^8" Harper et al^36^  “All she talked about was breast cancer and the risk of breast cancer. She mentioned it six times in a 10-minute conversation… she never once mentioned about the benefit of taking HRT” - Hobson & Dennis^41^ |
| Desire for HCP to discuss alternatives to HRT |  |  | Langer-shapland et al^26^, Morris & Symonds^27^, Aljumah et al^32^, Barber et al^34^, Harper et al^36^, Ray et al^38^ | "It would’ve been nicer to have a continued discussion because it is an ever-changing thing …and I don’t feel because of the timing I’ve had that opportunity." Barber et al^34^  ". . . it can be quite frightening without knowing why you’re feeling this way. More education for GP’s to have these conversations with women is necessary. Have they considered menopause clinics, support groups with in GP practices? #505" Harper et al^36^ |
| Seeing the doctor is a key step in the management of menopause symptoms |  |  | Adelekan-Kamara et al^19^ , Duffy et al^23^, Morris & Symonds^27^, |  |
| Mixed opinions in how doctors could support women |  |  | Duffy et al^23^, Prothero et al^28^, Langer-shapland et al^26^, Harper et al^36^ | "I don’t feel like a visit to the GP would help me, I would most likely get prescribed a generic HRT, no tests would be done and I’d be told ‘let’s see how you get on with this’. I’m planning to go privately to seek help for a more tailored approach rather than a trial and error method that NHS offers. #8^46^" Harper et al^36^ |
| Belief that the doctor cannot help for menopause, due to lack of time, ability and treatment options |  |  | Langer-shapland et al^26^, Morris & Symonds^27^, Utian & Maamari^29^, Barber et al^34^ | Many women did not seek help because they did not want to “waste a doctor’s time” or they thought the GP would not be able to do anything as “the menopause happens to everybody”. Barber et al^34^ |
| Alternative therapies, herbal remedies, vitamins and supplements used for menopause |  |  | Duffy et al^23^, Green et al^24^, Langer-shapland et al^26^, Morris & Symonds^27^, Whiley et al^31^ , Willis et al 2011^49^, Harper et al^36^, Ray et al^38^, Tanna et al^39^ | "Most literature I’ve seen seems to avoid alternative approaches or herbal alternatives. I have found the social media sites celebrating older women helpful in resetting my mindset and positive mental approach." Harper et al^36^; "I said “OK I'll try some other things” and I tried some red clover and all that and it didn't make a blind bit of difference, if anything it made me worse." Duffy et al^23^ |
| HRT helped with menopause symptoms |  |  | Aljumah et al^32^, Barber et al^34^, Harper et al^36^, Pearce et al^37^, Wasley & Gailey^40^ |  |
| Changing the environment to deal with symptoms |  |  | Langer-shapland et al^26^, Morris & Symonds^27^, | "I've got a big fan in the office and I have it facing me...I don't go on holiday in July or August any more, because it's too hot" (Anne) Morris & Symonds^27^, |
| Lifestyle changes seen as beneficial |  |  | Ballard et al 2002^21^, Duffy et al^23^, Langer-shapland et al^26^, Morris & Symonds^27^, Aljumah et al^32^, Barber et al^34^, Ray et al^38^ | “Some of the things that are really important are making more time for myself. So, more time to unwind, more time to go and do nice things- that self-care, like cold water swimming. That's been really helpful, you know. It's- it's those things that are going to kind of re-balance me you know. For me, nutrition and the supplement is really important. Ray et al^38^ |
| Unrealistic expectations of HRT |  |  | Barber et al^34^ | “see taking HRT as a thing that will fix that [problem in their life such as relationship breakdown], and it won’t”. Barber et al^34^ |
| Social media was an important source of information | **Media** | **Theme 2 – Understanding of menopause** | Aljumah et al^32^, Harper et al^36^, Ray et al^38^, Karavidas & Visser^46^, Willis2008^50^ | "Most literature I’ve seen seems to avoid alternative approaches or herbal alternatives. I have found the social media sites celebrating older women helpful in resetting my mindset and positive mental approach." Harper et al^36^  “I’ve had more information off [social media] than my doctor”. Karavidas |
| Frustration with the portrayal of menopause on media |  |  | Aljumah et al^32^, Ray et al^38^, Rubinstein & Foster^45^ | “I mean, I know Davina has done great things recently and this and that, but I feel like that's all about HRT, HRT. And my husband's like, ‘you need to get on HRT.’ I'm like, No. Well, I said, I'll research it…But now it's like, the only information we have is HRT.” [#^27^] Ray et al^38^ |
| Negative publicity with regards to HRT |  |  | Duffy et al^23^, Aljumah et al^32^, Harper et al^36^ | "The issues around HRT feel very politicised . . . I struggle to find any ‘neutral’ information about it. Articles are either very pro-HRT or very anti. #767" Harper et al^36^    "I think that all the recent publicity would have worried me, as it gives the impression that all women will have severe symptoms unless we take HRT – which is not the case for me or most of my friends.” Harper et al^36^ |
| Juxtaposition of the media portrayal of menopause and the actual ageing process |  |  | Ballard et al 2005^20^ | I think the menopause brings this getting older bit home to you. You know thisis happening and your body is changing, and you start thinking, oh gosh, I’vegot to do something about this. But attitudes are changing. I think it’s got a lotto do with this baby-boomer thing. The media help with this because they’reshowing all these beautiful older women and they’re saying, well look at her,she’s 50 and she has the figure she had when she was 20. I think this makespeople more aware that they’re not quite as old as they used to be at that age.” Ballard et al, 2005 |
| A celebrity menopause documentary was a useful information source |  |  | Aljumah et al^32^, Ray et al^38^ | “As to myself and perimenopause I really had no idea this was coming...but the first symptoms, the brain fog and the weird sleeping and suddenly getting hot and cold it was like, "what is this?" I-sounds incredibly naive, but I really didn't see it coming until I started kind of experiencing things myself and then good old Davina on the TV, and then talking to friends and realising okay, right I need to get my head around some of this stuff.” [#^22^] |
| Media (Books, magazines, media, local library and websites) used to learn about menopause |  |  | Morris & Symonds^27^, Aljumah et al^32^, Barber et al^34^, Ray et al^38^ |  |
| GPs had a negative view on media publicity on HRT |  |  | Barber et al^34^ | "One described feeling disbelief about media stories from women saying they have to “fight their GP” for HRT" Barber et al^34^ |
| UK Asian women had more vivid symptom descriptions | **Impact of culture and/or ethnicity** | **Theme 2 – Understanding of menopause** | Hunter et al^25^ | "I feel so hot that I feel like dying. I take off all the clothes (sweaters), strat fan and then after few minutes, I feel cold again" (UKA8) Hunter et al^25^ |
| Culture differences contributes to the taboo around menopasue |  |  | Adelekan-Kamara et al^19^ |  |
| UK Asian and UK Caucasian women reported emotional changes, though this was expressed differently. |  |  | Hunter et al^25^ | "Suddenly, while I am sitting, my face becomes really hot, it's like I am on fire and my mouth gets filled with water and I feel ghabrahat (palpitations and panicky feelings) and I feel as if I should take all clothes off and run" (UKA85) Hunter et al^25^ |
| UK Caucasian women more likely to talk about sexual symptoms than UK Asian women |  |  | Hunter et al^25^ |  |
| UK Asian women mentioned a lack of discussion regarding menopause. UK Caucasian women tended to speak to other women in their lives. |  |  | Hunter et al^25^ | "Anything to do with sex is hidden under the carpet in Indian culture. Like I am educated but had no idea about menopause. I didn’t talk to anybody. My doctor was Indian but didn’t say anything. It is some- thing about the Indian culture. We do suffer in silence" (UKA7) Hunter et al^25^ |
| Both UK Asia and UK Caucasian women mentioned that menopause did not bother them much |  |  | Hunter et al^25^ |  |
| UK Caucasian women tended to highlight reproductive status more |  |  | Hunter et al^25^ |  |
| UK Asian women tended to highlight increases feeling of cleanliness, and ability to partake in religious activities |  |  | Hunter et al^25^ | "Good for religious practice- can join in and read Namaj" (UKA14) Hunter et al^25^, |
| UK Asian women reported experiencing differing physical symptoms compared to UK Caucasian women |  |  | Hunter et al^25^ | "Your eyesight goes down when you stop having periods" (UKA52) Hunter et al^25^ |
| Asian women wanted more information from their GPs |  |  | Tanna et al^39^ |  |
| UK African and Asian women less likely to seek support for menopause |  |  | Barber et al^34^ | Not a quote "Both GPs and gynaecologists saw fewer women from African and Asian ethnic groups and believed that the possible explanations for this included: differences in the cultural significance and meaning of the menopause; differing degrees of medicalisation of the menopause; the expectation that women will follow intergenerational cultures; media coverage portraying the menopause as a white, middle-class issue; and inherent racial bias and language barriers in seeking medical help – especially when a friend or family member is the translator." Barber et al^34^ |
| Caucasian women felt the GP was the gatekeeper for their menopause care |  |  | Tanna et al^39^ |  |
| Caucasian women asked to actively participate in their management and care |  |  | Tanna et al^39^ |  |
| Women with cancer found it difficult to distinguish menopause symptoms from cancer symptoms | **Medical conditions and Menopause** | **Theme 3: Strategies to Manage Menopause** | Tanna et al^39^ |  |
| Greater acceptance and tolerance of menopause in women who have had cancer |  |  | Tanna et al^39^ | Not a proper quote -> Patients had an attitude of acceptance of menopausal symptoms, even when these were ‘difficult’ side-effects of prescribed breast cancer treatments such as TamoxifenRx, because the side-effects experienced from chemotherapy were ‘much more worse’ to cope with. Tanna et al^39^ |
| Lack of knowledge surrounding how menopause would affect pre-existing conditions |  |  | Harper et al^36^, Tanna et al^39^ | I have PCOS, there is practically no information available about PCOS and the menopause. I have kind of worked out for myself that my more regular periods might be a perimenopause symptom as my excessively high oestrogen levels fall into a more normal range. My GP has absolutely no idea about PCOS and menopause symptoms . . . I have no idea if there are any extra things I should be trying to manage as the PCOS and menopause interact. #690 Harper et al^36^ |
| GP could not answer questions relating to comborbidities and menopause |  |  | Barber et al^34^, Harper et al^36^ | Not a quote Gynaecologist reported.."In addition, they reported a growing number of referrals from GPs wanting specialist care for women with comorbidities such as obesity and gynaecological conditions." Barber et al^34^ |
| Symptoms negatively impacting pre-existing medical conditions |  |  | Brown et al^22^ , Harper et al^36^, Ray et al^38^ | "Further, a few women stated that their PMS symptoms were ‘ramped up’, causing more emotional distress and less patience with those around them." Ray et al^38^ |
| Autistic traits were heightened around menopause (emotional and sensory sensitivity) | **Menopause in other sub-groups** | **Theme 2 – Understanding of menopause** | McCarthy^43^, Karavidas & Visser^46^ | “they kept messing me about, I didn’t know where I was with them”. - McCarthy^43^  “I noticed a lot of very subtle things that most people wouldn’t”, such as changes in body hair. - Karavidas&Visser^46^  My body has all kinds of strange sensory reactions and um ... I don’t read it particularly well at the best of times ... It was just another thing that annoyed me about my body. - Karavidas & Visser^46^ |
| Difficulty coping with menopausal changes due to disruption in routine |  |  | Karavidas & Visser^46^ | It’s very disturbing and, you know, now I understand why actually. You think you know, ‘Four weeks, four weeks, four weeks’, and then you’re like ‘What! No, that’s not right!” - Karavidas & Visser^46^ |
| Misconceptions about menopause |  |  | Langer-shapland et al^26^, Willis2008^50^  Willis et al 2011^49^,  McCarthy^43^, Karavidas & Visser^46^ | “as you get older you can’t have sex because you can ’t have babies, because your body changes”- McCarthy^43^ |
| Loss of fertility associated with menopause not felt due to fertility not being "used" |  |  | McCarthy^43^ |  |
| Limited recognition of own self menopausal symptoms. Lack of understanding/recognition of their own menopausal status |  |  | Langer-shapland et al^26^, Willis2008^50^  Willis et al 2011^49^  McCarthy^43^  Karavidas & Visser^46^ |  |
| Wider lack of knowledge of female reproductive health |  |  | Willis2008^50^, Willis et al 2011^49^, McCarthy^43^ |  |
| Individuals would turn to their carers or female relative for support |  |  | Langer-shapland et al^26^, Willis2008^50^  Willis et al 2011^49^, Karavidas & Visser^46^ |  |
| Limited information from carers and family members around menopause |  |  | Langer-shapland et al^26^, McCarthy^43^, Karavidas & Visser^46^, Willis et al 2011^49^ | If I’d known about it before-hand I’d be able to say, ‘Oh yes, maybe the migraines were menopausal, maybe this was menopausal, maybe this was menopausal’, but it’s all passed me by because I didn’t know about the menopause. (Isla) - Karavidas & Visser^46^ |
| Preference for simple book/booklet to read at home |  |  | Willis et al 2011^49^, Karavidas |  |
| uncertanity and conflulation of menopause with general signs of ageing, stresses and other causes |  |  | Langer-shapland et al^26^, McCarthy^43^ |  |
| Information wanted to help others |  |  | Langer-shapland et al^26^, | "Then I can understand if somebody else asks me that I could help them" (Jane) Langer-shapland et al^26^, |
| Time needed to process information |  |  | Langer-shapland et al^26^, | "After she explained it....I thought it was gonna tak me a few days to register this one in" (Jane) Langer-shapland et al^26^, |
| Belief that because you have not had children, you cannot understand menopause |  |  | McCarthy^43^ |  |
| Suggestions for support: Wait for symptoms ot pass, speak to doctor |  |  | McCarthy^43^, Karavidas & Visser^46^ | “With autistic girls you need to plan for puberty way before puberty. It's the same with ageing and menopause”. These quotes suggest that menopause would be more tolerable if participants knew what to expect. Karavidas & Visser^46^ |
| Normalisation of menopause |  |  | McCarthy^43^ |  |
| Temporary staff led to disjointed care |  |  | Karavidas & Visser^46^ |  |
| absence of professional awareness of the healthcare and communication needs of autistic adults |  |  | Karavidas & Visser^46^ |  |
| Dissociation as a coping mechanism |  |  | Karavidas & Visser^46^ | I worry sometimes, ‘What have I become?’… what's the word? Have I become dissociated? I can’t cry because I'm dissociated. I don't know. But I don't cry much now, hardly ever.    Karavidas & Visser^46^ |
| Autism diagnosis gave confidence and entitilement to seek support |  |  | Karavidas & Visser^46^ | “It's not up to me to try and cover it up”. Similarly, Grace commented: “I’m brilliant at masking and actually, some of the time I really rather enjoy it… the question is whether I choose to… anymore”. Karavidas |
| Inability to access a healthcare professioanl of their choice (female) |  |  | Langer-shapland et al^26^, Willman & King^48^ , McCarthy^43^, Willis et al 2011^49^ | GA: ... as long as it is a lady doctor, because a man couldn’t explain to a woman, like a woman can. MM: You think it should be a lady doctor? GA: Yes, because a man doctor might tell them the wrong thing - McCarthy^43^ |
| Lack of routine Female health checks disincentivised from seeking help |  |  | Willman & King^48^ |  |
| Cessation of periods associated with increased ease of social engagements |  |  | Langer-shapland et al^26^ | "I've been really bad with it before where I was going somewhere and I just couldn't go out with my friends" (Helen) Langer-shapland et al^26^; "easier....'cause I can do things" (Sunshine" Langer-shapland et al^26^ |
| Improvement of mood secondary to cessation of periods in those with learning disabilities |  |  | Langer-shapland et al^26^ | "Happier" (Sunshine) Langer-shapland et al^26^, |

*Table S4: A table to show all primary codes extracted from the included studies and illustrative quotes.*
